# Field‐Effect Transistors from Artificial Charged Domain Walls in Stacked Van der Waals Ferroelectric α‐In_2_Se_3_

**DOI:** 10.1002/adma.202523096

**Published:** 2026-01-30

**Authors:** Shahriar Muhammad Nahid, Haiyue Dong, Gillian M. Nolan, SungWoo Nam, Nadya Mason, Pinshane Y. Huang, Arend M. van der Zande

**Affiliations:** ^1^ Department of Mechanical Science and Engineering, Grainger College of Engineering University of Illinois Urbana–Champaign Urbana USA; ^2^ Department of Physics, Grainger College of Engineering University of Illinois Urbana–Champaign Urbana USA; ^3^ Department of Materials Science and Engineering Grainger College of Engineering University of Illinois Urbana–Champaign Urbana USA; ^4^ Department of Mechanical and Aerospace Engineering, Department of Materials Science and Engineering University of California, Irvine Irvine USA; ^5^ Pritzker School of Molecular Engineering University of Chicago Chicago USA; ^6^ Department of Materials Science and Engineering Materials Research Laboratory Grainger College of Engineering University of Illinois Urbana–Champaign Urbana USA; ^7^ Department of Mechanical Science and Engineering, Department of Materials Science and Engineering, Materials Research Laboratory, Grainger College of Engineering University of Illinois Urbana–Champaign Urbana USA

**Keywords:** α‐In_2_Se_3_, charged domain walls, electrical transport, heterostructure, van der waals ferroelectric

## Abstract

Ferroelectric charged domain walls (CDWs) offer emergent electronic states that can serve as functional elements in high‐density nonvolatile memory and neuromorphic computing. Yet, poor conductivity, structural instability, and lack of deterministic control limit their practical use. Moreover, the CDWs are typically out‐of‐plane and buried interfaces, which prohibits electrical access and prevents gate control of their carrier density. This work demonstrates the fabrication of artificial in‐plane CDWs by stacking oppositely polarized flakes of van der Waals (vdW) ferroelectric α‐In_2_Se_3_. Edge contact is utilized to electrically access the CDWs and integrate them into CDW‐based field‐effect transistors (CDW‐FETs). CDW‐FETs exhibit room‐temperature conductance up to four orders of magnitude higher than single domains, exceeding previously reported CDWs by 2–9 orders of magnitude. Electron microscopy imaging reveals atomic reconstruction and interfacial heterogeneity in CDWs. Temperature and gate‐dependent electrical and magneto‐transport measurements confirm that interfacial band bending governs transport. Two transport mechanisms are identified in these CDW‐FETs: variable‐range hopping and thermally activated traps, showing a transition temperature of 80 K. These results establish artificial CDWs as on‐demand, designable conductive channels in vdW ferroelectrics, advancing the understanding of CDW conduction mechanisms and bridging the gap toward device integration.

## Graphical abstract

This study utilizes the van der Waals stacking of ferroelectric α‐In_2_Se_3_ to fabricate in‐plane artificial charged domain walls. These charged domain walls are electrically accessible, gate‐tunable, and show 2–9 orders of magnitude higher conductance than charged domain walls from thin film ferroelectrics, allowing their integration in field‐effect transistors. Atomic scale imaging and temperature‐dependent transport confirm that interfacial heterogeneity governs their electronic transport.

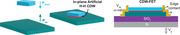

## 1 Introduction

The quest for energy‐efficient and reconfigurable electronics has driven an intense interest in material platforms that go beyond conventional semiconductors. Ferroelectrics, with their intrinsic bistability and coupled electronic, structural, and polar degrees of freedom, are prime candidates for low‐power memory, neuromorphic computing, and adaptive circuits. Ferroelectric domain walls occur at the interface between regions of different polarization that can act as functional nanoscale elements distinct from the adjacent bulk domains [1, 2, 3, 4]. Over the past two decades, domain walls have been shown to host interfacial properties such as enhanced conductivity [5], emergent polarity [6], magnetic order [7, 8], and enhanced electromechanical coupling [9, 10]. Of particular interest are charged domain walls (CDWs), where domains with opposite polarization create interfacial bound charges that require screening by free carriers [11]. Consequently, CDWs exhibit orders of magnitude higher conductivity compared to the surrounding domains [1, 2, 3, 5] and even host 2D electron and hole gasses [12, 13]. These exciting properties of CDWs lead to potential applications in nonvolatile memory and artificial synapses [1, 2].

However, realizing such visions of CDW‐based devices in thin‐film perovskite ferroelectrics remains a challenge. First, CDWs are not well controlled and are typically randomly self‐assembled with meandering or discontinuous paths [2]. Second, CDWs are frequently oriented along the out‐of‐plane direction [5, 12, 14] or buried in the bulk [15]. Third, CDWs exhibit MΩ‐TΩ resistance, as measured by conductive atomic force microscopy or vertical transport techniques [5, 12, 16, 17]. These values are orders of magnitude too high for most technologically relevant applications. Other studies demonstrate mobile CDWs under out‐of‐plane electric fields but cannot electrically access these buried CDWs to separate their conduction from the bulk [15]. There is a need for new strategies to create in‐plane, electrically accessible, and gate‐tunable CDWs that can be integrated into devices, as well as a more complete understanding of the mechanisms and limits of interfacial conduction.

The emergence of van der Waals ferroelectrics provides an opportunity to reimagine domain wall‐based electronics. Due to their layered structure, these materials enable clean stacking and precise control over interface geometry, allowing artificial polarization configurations that are difficult to stabilize in bulk crystals [18, 19]. Moreover, van der Waals ferroelectrics show fundamentally different phenomena including stable polarization in the monolayer limit [20], quadruple well potential [21], metallic ferroelectricity [22], bending angle dependent polarization reversal [23], enhanced photovoltaic effect [24], or polarity from nonpolar parent materials [25]. An unexplored possibility is that van der Waals ferroelectric interfaces can provide access to a new class of in‐plane CDWs with new knobs to program their properties.

In this work, we report the creation of artificial CDWs from α‐In_2_Se_3_ heterostructures and their integration into CDW‐based field‐effect transistors (CDW‐FETs). By stacking two multilayer flakes of α‐In_2_Se_3_ with opposite polarization, we generate continuous head‐to‐head (H‐H) CDWs that exhibit conductance up to four orders of magnitude higher than single domains and 2–9 orders of magnitude higher than previously reported ferroelectric CDWs. Atomic scale imaging reveals continuous nanometer scale walls with interfacial heterogeneity, including regions of nonpolar β‐In_2_Se_3_, amorphous material, and nanogaps. Temperature and gate‐dependent transport measurements establish band bending‐induced conduction, modulated by carrier hopping and trap‐assisted processes. These results demonstrate the highest‐performing CDWs to date and establish a general framework for engineering ferroelectric domain walls as on‐demand, high‐conductivity, and gate‐tunable channels. In doing so, our work bridges a long‐standing gap between domain wall physics and practical nanoelectronic applications.

## 2 Results and Discussion

### 2.1 Enhanced Conductivity in Artificial CDW‐FETs

**FIGURE 1:**
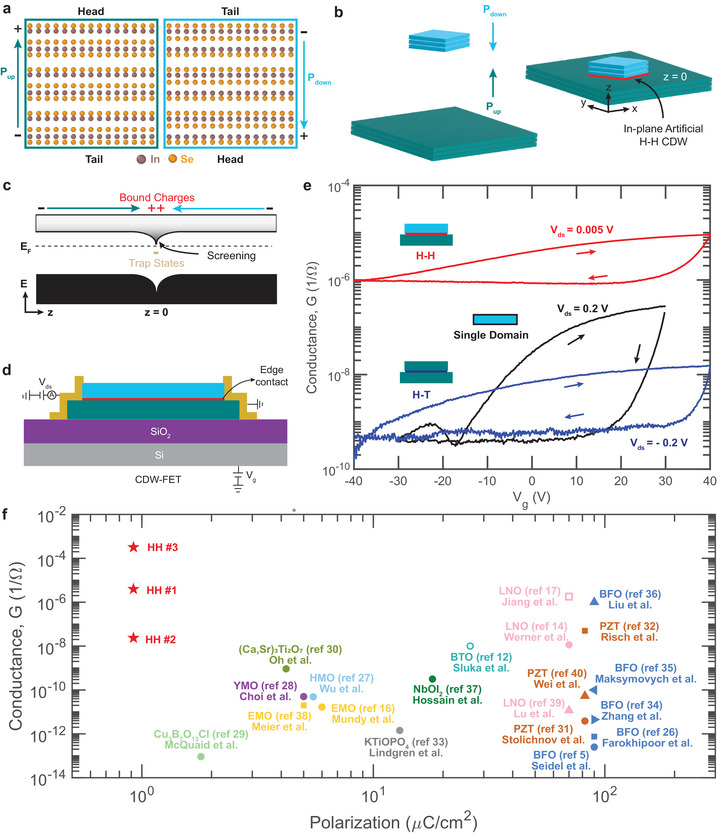
Structure and properties of charged domain wall field‐effect transistors (CDW‐FETs). (a) Atomic structure of van der Waals ferroelectric α‐In_2_Se_3_ showing the two out‐of‐plane polarizations. $P_{up}$ (teal) and $P_{down}$ (cyan) arises from the displacement of central Se atom in each unit cell. The heads and tails of the polarized layers have positive and negative bound charges, respectively. (b) Schematic of an in‐plane artificial head‐to‐head (H‐H) CDW (red) formed by incommensurately stacking two oppositely polarized multilayer flakes. (c) Out‐of‐plane band diagram showing band bending at the H‐H CDW due to the positive bound charges. Tan block shows thermally activated interfacial trap states. (d) Schematic of a CDW‐FET. Source and drain electrodes make edge contacts to the CDW, while the silicon substrate acts as a global back gate. (e) Comparison of the room‐temperature conductance versus gate bias curves of a H‐H CDW‐FET (red), head‐to‐tail (H‐T) heterostructure FET (blue), and single domain α‐In_2_Se_3_ FET (black). (f) Comparison of the room‐temperature conductance of CDW‐FETs with other ferroelectric domain walls [5, 12, 14, 16, 17, 26, 27, 28, 29, 30, 31, 32, 33, 34, 35, 36, 37, 38, 39, 40]. The α‐In_2_Se_3_ H‐H CDWs (H‐H #1, #2, and #3) are marked by red stars. H‐H #3 has the highest conductance of $3.2\times 10^{-4}$ $\Omega^{-1}$, which is 2 to 9 orders of magnitude higher than previous reports.

Figure 1 shows how we create CDW‐FETs from α‐In_2_Se_3_ heterostructures. As shown in Figure 1a, α‐In_2_Se_3_ is a van der Waals ferroelectric, where the displacement of the central atomic plane of Selenium within each unit cell leads to out‐of‐plane polarization [41]. The polarization vector points from negative charge (tail) to positive charge (head). Figure 1b graphically shows how we form incommensurate H‐H CDWs by using aligned transfer to stack two multilayer α‐In_2_Se_3_ flakes with polarizations pointed toward each other. Piezoelectric force microscopy (PFM) confirms the relative polarization of each flake and the final heterostructure (Figure S1). See Experimental Section for details on heterostructure fabrication and PFM characterization. Section S1 discusses the relative yield of the four possible unique heterostructures formed by this process. Figure 1c shows the proposed out‐of‐plane (z) band diagram of the H‐H CDW, which governs in‐plane (x‐y) transport. The local electrostatic potential of the positive bound charges results in a downward band bending at the CDW, bringing the conduction band minima closer to the Fermi energy ($E_{F}$) and allowing free electrons to screen the bound charge. Additionally, disorder at the interface leads to trap states, shown as the tan block.

Figure 1d shows the schematic of a CDW‐FET, fabricated from a H‐H CDW (see Experimental Section). Electrical access to the CDW is achieved through edge contact (Figure S2), similar to the techniques employed in 2D material heterostructures [42]. The SiO_2_/Si substrate serves as the global back gate. Figure 1e compares the conductance versus gate bias curves at 300 K of a H‐H CDW‐FET (labeled H‐H #1) with a head‐to‐tail (H‐T) heterostructure FET and a single domain FET, including upward and downward sweeps. The H‐T and single domain structures serve as controls to isolate the impact of the H‐H CDWs from the effects of stacking‐transfer (H‐T) and bulk effects in structures lacking domain walls. Figures S3 and S4 show the corresponding output and gate leakage measurements. Table S1 summarizes the dimensions of each FET.

Both single domain α‐In_2_Se_3_ and H‐T heterostructure FETs display n‐type semiconductor transport, consistent with the literature [43]. In contrast, the CDW‐FET shows drastically higher conductance at all gate biases, consistent with the proposed band diagram. At 30 V gate bias, the CDW has conductance 30x that of the single domain and 600x that of the H‐T heterostructure. Moreover, the CDW‐FET shows less than 1 order of magnitude modulation in conductance under gating. Importantly, the H‐T heterostructure does not show enhanced conductance, indicating that the presence of a stacked interface alone is insufficient to explain the high conductance of CDW‐FET. We confirm the robustness of the conductance by characterizing two additional CDW‐FETs, labeled H‐H #2‐3, with PFM and transport shown in Figures S5 and S6. All results in the main text are from H‐H #1.

**FIGURE 2:**
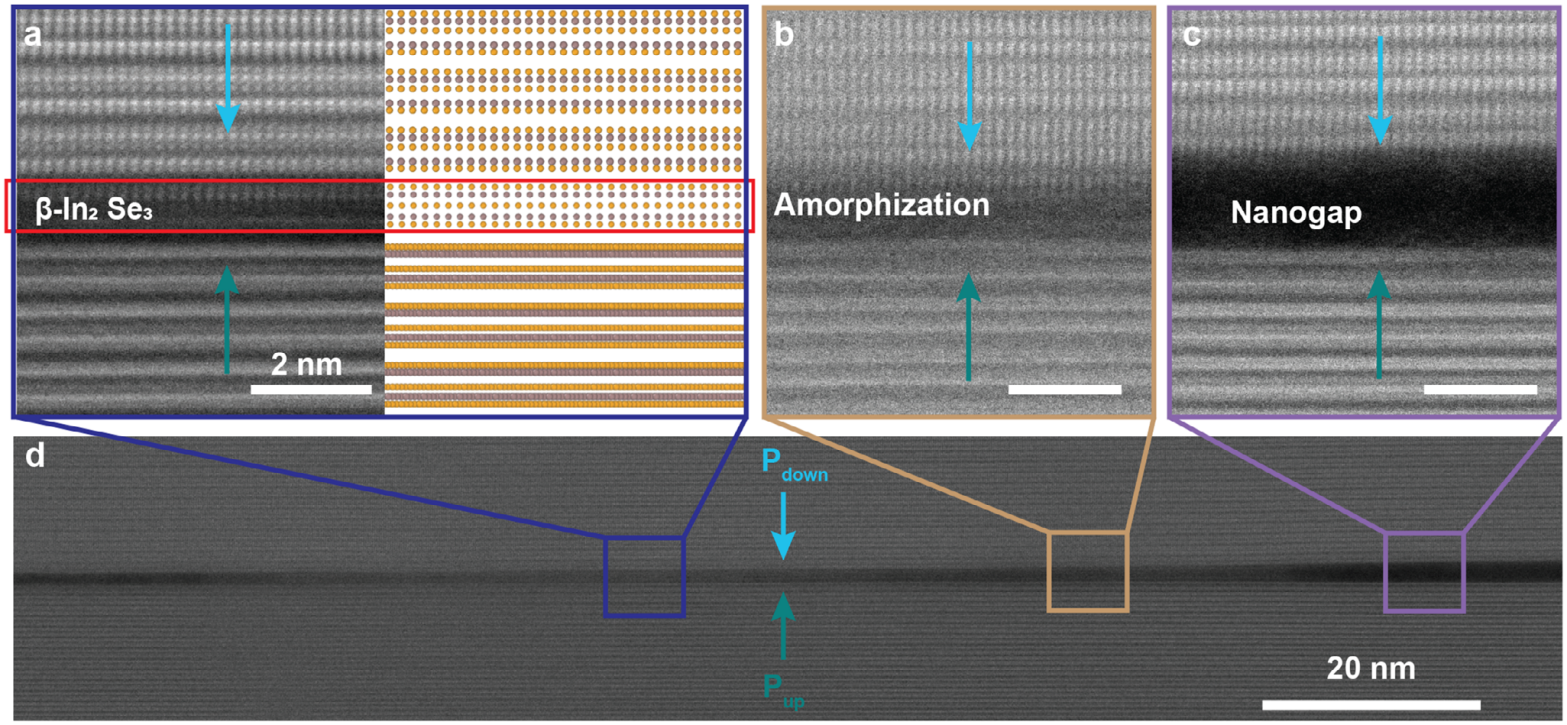
Atomic structure of the H‐H CDW in α‐In_2_Se_3_. (a–c) Cross‐sectional STEM images of the atomic structure in different regions of H‐H CDW (H‐H #1). (a) shows the cross‐sectional STEM image and the corresponding atomic structures for comparison. Each region has uniform polarization domains in the top and bottom flake. However, the CDW exhibits interfacial heterogeneity including (a) the formation of β‐In_2_Se_3_ phase (red rectangle), (b) amorphization of α‐In_2_Se_3_, and (c) nanogap. (d) Low‐magnification image of the H‐H CDW showing the relative location of each region.

**FIGURE 3:**
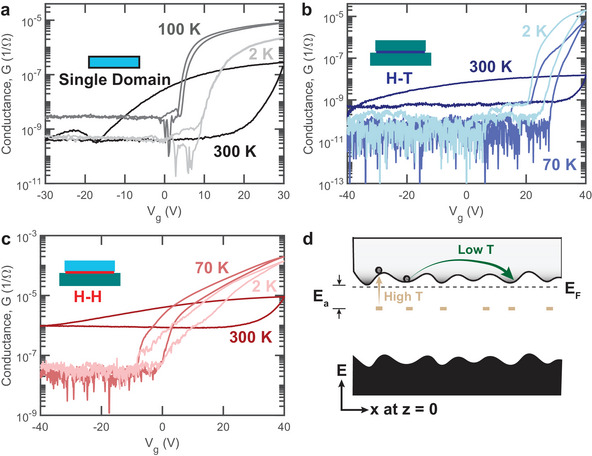
Temperature‐dependent electrical transport of the CDW‐FET. Conductance versus gate bias transfer curves of (a) single domain, (b) H‐T heterostructure, and (c) CDW‐FET at key temperatures. (d) The proposed in‐plane band diagram of the CDW. Interfacial heterogeneity results in in‐plane modulation of the band edges and generates distributed trap states (marked as tan blocks) with an activation energy $E_{a}$. At low temperature, carriers travel through the modulated band edges, while at high temperature, transport occurs through activated trap states.

**FIGURE 4:**
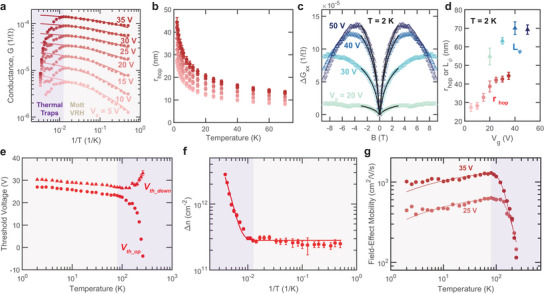
Analysis of temperature scaling and magnetotransport of the H‐H CDW. (a) Conductance versus inverse temperature (1/T) extracted from the up‐sweep at gate bias from 5 to 35 V (pink to red points). Solid lines are fits showing the scaling behavior predicted by the VRH model for temperatures < 80 K. (b) Hopping length ($r_{hop}$) versus temperature at different gate biases derived from the VRH model. (c) Change in low‐temperature (T= 2 K) magneto‐conductance ($\Delta G_{xx}$) with magnetic field (B) for gate biases from 20 to 50 V (light to dark blue). Solid lines show the low field |B|<3 T fit to the HLN model for weak localization. (d) Comparison of the low‐temperature (T= 2 K) derived phase coherence length ($L_{\phi }$, blues), and hopping length ($r_{hop}$, reds) versus gate bias. (e) Threshold voltage for both up‐sweeps ($V_{th\_up}$, triangles) and down‐sweeps ($V_{th\_down}$, points), extracted from the temperature dependent transfer curves of CDW‐FET. (f) Derived trap density (Δn, points) versus inverse temperature 1/T calculated from the threshold voltage difference between up‐ and down‐sweeps. Solid line is fit to Arrhenius relation for thermally activated trap density. (g) Field‐effect mobility versus temperature. Values are derived from the up‐sweep at 25 and 35 V gate bias. The solid line shows the fit, which considers both the VRH and thermally activated traps according to the Matthiessen's rule. The shades in panels a,e,f, and g denote the transport mechanisms dominating at different temperatures. All the error bars represent 95 % confidence interval.

The enhanced interfacial conductance in the CDW‐FETs are analogous to polar discontinuities in out‐of‐plane CDWs in perovskite ferroelectrics as well as epitaxially grown piezoelectric or polar heterointerfaces [1, 4, 44, 45]. To quantify performance, Figure 1f is a scatter plot of the room‐temperature conductance versus polarization, comparing H‐H #1‐3 (red stars, extracted at zero gate bias) with published values for out‐of‐plane ferroelectric CDWs [5, 12, 14, 16, 17, 26, 27, 28, 29, 30, 31, 32, 33, 34, 35, 36, 37, 38, 39, 40]. The shape represents different studies, while the color represents different ferroelectric materials. Because only the out‐of‐plane CDWs in thin‐film ferroelectrics are electrically accessible, conductance is typically measured with two‐point measurements via conductive atomic force microscopy (CAFM) probe‐based techniques (denoted as solid markers) [5, 14, 16, 26, 27, 28, 29, 30, 31, 32, 33, 34, 35, 36, 37, 38, 39, 40] or by patterned top electrodes (hollow markers) [12, 17]. The best comparison to CDW‐FETs in this study is patterned electrodes because they have a channel length‐to‐contact width ratio of 0.3‐1, comparable to the in‐plane CDWs in this study. The CDW conductance depends on the free carrier density at the interface, which depends on ferroelectric polarization, bandgap, and mobility, as well as the continuity of the domain wall. In general, there is no clear correlation between CDW conductance and host material bandgap, which ranges from 1.4 to 3.8 eV. Meanwhile, as seen in Figure 1f in most thin‐film ferroelectrics, CDW conductance is correlated with polarization, with higher polarization leading to higher conductance, although there is enormous variation even between studies on the same materials [4]. Surprisingly, despite α‐In_2_Se_3_ having the lowest polarization and a moderate bandgap of 1.4–1.6 eV [43, 46], the CDW‐FETs show conductance that ranges from on par to 2 orders of magnitude higher than the best reported values for out‐of‐plane CDWs. While this comparison involves different measurement geometries, such as lithographically defined micron‐scale edge contacts versus nanoscale CAFM point contacts, even conservative considerations of geometric factors confirm that the comparison is robust to differences in measurement geometry (Table S2). Section S2 discusses these geometric effects and their limitations. In particular, H‐H #3 shows the highest conductance of $3.2\times 10^{-4}$ 1/Ω (resistance 3.1 kΩ), comparable to the values observed in epitaxial piezoelectric interfaces [44, 45, 47]. However, the three CDW‐FET samples, H‐H #1‐3, have nearly 4 orders of magnitude differences in conductance, showing that the performance is not purely from material properties. From these trends, we hypothesize that the high conductivity in the CDW‐FETs arises from a fundamentally different disorder and lower scattering at the van der Waals interface in comparison to thin‐film ferroelectrics. To understand how the van der Waals interface defines CDW disorder and transport mechanisms, Figures 2, 3, 4 relate the atomic structure with temperature, gate bias, and magnetic‐field‐dependent electrical transport of the CDW‐FET.

### 2.2 Atomic Structure of Artificial CDWs

Figure 2 examines the atomic structure of the CDW. After transport measurements, we image the CDW‐FET interface using aberration‐corrected annular dark‐field scanning transmission electron microscopy (ADF‐STEM) (Experimental Section). Figure 2a–c show cross‐sectional STEM images of different regions of the CDW, while Figure 2d shows the relative location of each region. Figure 2a includes the corresponding schematic of the crystal structure. The top and bottom flakes have down and up polarization, respectively, creating an H‐H CDW at the interface (red rectangle). Due to incommensurate stacking, atomic columns are aligned in the top flake, whereas only the atomic planes are discernible in the bottom flake.

There are three important observations. First, atomic reconstruction occurs at the H‐H CDW interface, which accommodates the interfacial polar discontinuity. Similar reconstruction is commonly observed in conventional ferroelectric CDWs [48]. Second, the CDW is 1–3 nm wide along the out‐of‐plane and remains continuous on micrometer length scales. In contrast, naturally occurring CDWs often shift between different atomic planes, resulting in discontinuous or meandering interfaces [48, 49, 50].

Third, the interface structure is heterogeneous. The different regions, shown in Figure 2a–c, consist of a monolayer β‐In_2_Se_3_ phase (Figure 2a), amorphous material (Figure 2b), or a nanogap (Figure 2c). The β‐In_2_Se_3_ phase, amorphization, and nanogaps are discernible by the position of the middle Se plane in the layer, the loss of crystallinity, and the negligible signal intensity between the flakes, respectively. Figure S7 shows other regions with additional features. Formation of monolayer β‐In_2_Se_3_ is consistent with the natural H‐H CDW observed in exfoliated α‐In_2_Se_3_ and is predicted to have new bands compared to the bulk [50, 51]. Since the thickness of the amorphous region is similar to the monolayer α‐In_2_Se_3_, we hypothesize that the amorphous region is α‐In_2_Se_3_ with reduced crystallinity. The nanogaps result from voids formed by bending at the step edges in the flakes' surfaces (Figure S7) [23, 52]. Figure 2d shows that these different features coexist in proximity with in‐plane separation of 5–10s of nm.

### 2.3 Transport Mechanisms of Artificial CDW‐FETs

Next, we performed temperature‐dependent electrical transport measurements to investigate how interfacial heterogeneity impacts transport. Figure 3 compares the temperature‐dependent transfer curves of FETs from a (a) single domain α‐In_2_Se_3_, (b) H‐T heterostructure, and (c) H‐H CDW. At 300 K, both single domain α‐In_2_Se_3_ and the H‐T heterostructure show n‐type semiconductor transport, while the CDW‐FET displays transport similar to degenerately doped semiconductors with significantly higher conductance and less than one order of magnitude modulation in conductance under gate bias. With decreasing temperature, all FETs show n‐type transport. At higher gate bias, the conductance of single domain and CDW‐FET increases from 300 K to a transition temperature, and then decreases. The CDW‐FET maintains enhanced conductance than the H‐T and single domain FETs at all temperatures. All these observations are consistent with the polar discontinuity‐induced interfacial band bending demonstrated in Figure 1c. Free carriers accumulate at the CDW due to band bending, enhancing the conductance and preventing significant gate modulation. Section S3 rules out alternative conduction mechanisms including stoichiometric variation or defect metallization.

All FETs show significant hysteresis at 300 K. However, at low temperature, the hysteresis in the single domain nearly vanishes, while the H‐T and CDW‐FET exhibit reduced but persistent hysteresis, indicating potential interfacial trap states independent of the interface type. Section S4 rules out macroscopic polarization switching as the origin of hysteresis, addresses possible minor microscale effects under high gate bias, such as defect dipole reorientation or localized domain wall motion, and identifies charge trapping as the primary origin of hysteresis.

Figure 3d shows the proposed in‐plane band diagram of the CDW. Heterogeneity in the CDW leads to spatially varying band structures and localized trap states, resulting in an in‐plane modulation of the band edges. Section S5 and Figure S8 provide a detailed discussion of how each structure modifies the local band diagram and trap state distribution. This structural heterogeneity contributes to the large device‐to‐device variations in CDW‐FET performance observed in Figure 1f and discussed further in Section S6.

The proposed band diagram explains the temperature‐dependent transport as a combination of two mechanisms, both of which are commonly observed in 2D materials with disorder [53, 54, 55]. First, the modulation of the band edges and bound charge density results in electron hopping at low temperature, as described by the Mott variable range hopping (VRH) [56, 57]. Second, localized states lead to thermally activated trap states (tan blocks) with an activation energy $E_{a}$, which induce temperature‐dependent hysteresis [54].

In Figure 4, we analyze temperature‐dependent transport and low‐temperature magnetotransport in terms of VRH and trap‐assisted transport models. Section S7 describes the governing equations of each theory. Figure 4a plots the conductance of CDW‐FET H‐H #1 versus inverse temperature (1/T) at 5–35 V gate biases, with drain bias $V_{ds}=5$ mV. At all gate biases, the conductance increases with increasing temperature to 80 K and then decreases. The transition suggests that different mechanisms dominate the low‐ and high‐temperature transport, indicated by the shades in the plot.

The low‐temperature scaling of the conductance is consistent with the VRH model. We fit the conductance to the VRH model up to 80 K, shown as the solid lines in Figure 4a. Figure 4b shows the corresponding mean hopping distance $r_{hop}$ versus temperature.

As an independent probe of disorder, we also perform magnetotransport measurement at 2 K. Figure 4c shows the longitudinal symmetrized conductance change (ΔGxx) versus magnetic field (B) of H‐H #1 for 20–50 V gate biases. The data show no Landau oscillations. The initial increase in ΔGxx with the magnetic field confirms the weak localization in this system. We fit the magnetotransport data using the Hikami–Larkin–Nagaoka (HLN) model [58] up to 3 T, as shown by the solid lines in Figure 4c. The fits provide the phase coherence length $L_{\phi }$ of the electrons in the system. Figure 4d compares $r_{hop}$ (red tone) with $L_{\phi }$ (blue tone) at T = 2 K for different gate biases. Both parameters are related to the length scale of the disorder, but are not identical. As is commonly observed, both parameters increase with increasing gate, corresponding with decreasing localization for higher energy electrons compared with the band modulations.

Next, we assess the hysteresis in the transfer curves in terms of trap density. Figure 4e plots the threshold voltage of the CDW‐FET, calculated from linear extrapolation, versus temperature for the up‐ ($V_{th\_up}$) and down‐ ($V_{th\_down}$) sweeps. Figure 4f plots the corresponding trap density (Δn) versus 1/T, calculated from the threshold voltage differences. At low temperature, $V_{th\_up}$ and $V_{th\_down}$ shift together and then diverge above a transition temperature of 80 K. The resulting trap density shows an exponential decay versus $T^{-1}$ for T<80 K, and constant for T>80 K. From fitting to the Arrhenius equation (Section S7), we obtain the thermally activated trap density $n_{thermal}=3.94\pm 1.94\times 10^{13}$ cm^−2^), the activation energy $E_{a}=64.6\pm 10.3$ meV, and the shallow trap density (small activation energy compared to the measured temperatures) $n_{shallow}=2.63\pm 0.44\times 10^{11}$ cm^−2^.

Finally, Figure 4g plots the field‐effect mobility versus temperature for CDW‐FET derived from the up‐sweep for 25 and 35 V gate biases. Figure S9 shows the complementary temperature scaling of the extracted subthreshold swing and threshold voltage difference. Here, we assume a uniform current density to estimate the mobility, but the non‐uniform shape of the top flake makes it likely that the current distribution is non‐uniform. As a result, the reported values represent the lower limits of mobility. The mobility shows a non‐monotonic trend with temperature, in accordance with the Mott VRH and trap‐assisted transport mechanisms described above (solid line fits). The fit yields $E_{a}=73\pm 6$ and 62±5 meV for 25 and 35 V gate biases, consistent with the value obtained from the hysteresis analysis. At gate bias = 35 V and T = 80 K, the mobility reaches a maximum of 1113±7 cm^2^ V^−1^ s^−1^. At 300 K, the maximum mobility is 16 and 218 cm^2^ V^−1^ s^−1^ for up‐sweep and down‐sweep condition. As shown in Figure S6, H‐H #3 shows higher mobility at 300 K of 523 and 898 cm^2^ V^−1^ s^−1^ for up‐sweep and down‐sweep respectively.

As a comparison, Figure S10 shows that the field‐effect mobility of CDW‐FET (H‐H #1 or 3) are 1–3 orders of magnitude higher than the single domain and H‐T sample at all temperatures, 1–4 orders of magnitude higher than the Hall mobility of the 2D carrier gas in LaAlO_3_‐SrTiO_3_ (0.1–10 cm^2^ V^−1^ s^−1^) [44], and are comparable to AlGaN/GaN (20‐834 cm^2^ V^−1^ s^−1^) [45, 59] and ZnMgO/ZnO heterostructures (200–300 cm^2^ V^−1^ s^−1^) [60].

Together, Figure 4 confirms the two transport mechanisms in CDW‐FETs: VRH at low temperatures and trap‐assisted transport at high temperatures. The transition temperature depends on the competition between the activation energy of the trap states and the band edge roughness. While the data are consistent with these two transport mechanisms, other effects, such as polarization or phase stability at the CDW, can play minor roles in transport and provide an interesting avenue for future studies.

## 3 Conclusion

In summary, we demonstrate a method for generating highly conducting ferroelectric CDWs on demand and use them to realize decades‐old predictions for a new class of CDW‐FETs. By controlling the carrier density via electrostatic gating, this work uncovers the role of nanoscale disorder and the mechanisms of conduction. The CDW‐FETs in this work uniquely combine and extend the key advantages of both ferroelectric CDWs and epitaxial polar heterointerfaces: the conductance is two orders of magnitude higher than the best reported values of out‐of‐plane CDWs, thus overcoming a key limitation of CDWs in high speed circuity applications [2, 36], while van der Waals stacking produces atomically sharp interfaces without chemical intermixing found in epitaxial interfaces [61]. Moreover, despite the already superlative properties, the structural and transport characterization suggests that there is further room for improvement in performance by improving the uniformity of the interface.

Beyond fundamental insights, these devices define a clear technological niche in CDW‐based memristors and memtransistors, where the CDW conductance can be actively tuned by reversibly switching the polarization of the constituent domains. As a result, a heterostructure containing *n* domains with *P* number of distinct polarizations can access $P^{n}$ unique states. This exponential scaling of conductance states will enable multibit and high‐density synaptic elements that are difficult to achieve with conventional memristors. In addition, the CDWs can serve as a multilevel memory component, potentially outperforming conventional ferroelectric memory in speed and power.

Finally, while demonstrated here with α‐In_2_Se_3_, this method of creating artificial CDWs should be universally applicable to any van der Waals ferroelectric with out‐of‐plane polarization. Such artificial CDWs can be flexibly designed by changing the material combinations or orientation. Meanwhile, the intrinsic instability of CDWs opens the door to new kinds of reconfigurability that cannot be accessed in epitaxial interfaces. Thus, these results represent a significant step forward toward the ultimate promise of CDWs as functional elements in nonvolatile memory and neuromorphic computing.

## 4 Experimental Section

### 4.1 Fabrication of Ferroelectric Heterostructures and Charged Domain Walls

We adapted a dry transfer technique, commonly used to make 2D material heterostructures [62], to fabricate the H‐H CDWs. We used a microscope transfer station inside a nitrogen glove box to prevent oxidization of α‐In_2_Se_3_ and ensure a clean interface. Inside the glovebox, we first mechanically exfoliated α‐In_2_Se_3_ flakes on SiO_2_ and polydimethylsiloxane (PDMS) substrates. We selected one flake from each substrate with the criterion that the flake on SiO_2_ was larger than the flake on PDMS. This ensured that in the final heterostructure, the flake on top did not fully cover the flake on the bottom, allowing electrical access to the interface. Then, using the transfer station, we lowered the flake on PDMS to slowly approach and contact the flake on SiO_2_. After contact, we heated the SiO_2_/Si substrate to 60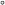 C for five minutes. Next, we slowly delaminated the PDMS, leaving a heterostructure on the SiO_2_ substrate. Finally, we removed the sample from the glovebox and performed PFM measurement in air to identify the heterostructure type (head‐to‐head, tail‐to‐tail, head‐to‐tail, tail‐to‐head) and selected the structures for subsequent field‐effect transistor (FET) fabrication and transport measurements.

### 4.2 Fabrication of Field‐Effect Transistors

We fabricated the CDW‐FETs using electron‐beam lithography to pattern the electrodes, ebeam evaporation to deposit the metal, and lift‐off. We started with single domain, H‐T heterostructure, and H‐H CDW heterostructures on 285 nm SiO_2_ on degenerately doped silicon. We defined a double layer resist with 495 A4 Poly (methyl methacrylate) (PMMA) and 950 A2 PMMA (first layer: spincoating 3000 rpm for 60s, bake at 110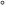 C for 20 min and Second layer: spincoating at 3000 rpm for 60 s, bake at 110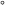 C for 25 min). Next, we used a Raith EBPG5150 Electron Beam Lithography System to write the electrode patterns (current 20 nA, aperture 300 nm, dose 800 µC cm^−2^). The design ensured the electrodes draping over the edge of the top flake, establishing edge contact with the CDW at the interface between the two flakes. We developed the PMMA by using a cold IPA and DI water mixture (volume ratio 3:1). We used ebeam evaporation to deposit 5 nm Ti, 60 nm Au, 10 nm Pd, and 60 nm Au. Finally, we performed lift‐off of the electrodes in acetone and subsequently cleaned the sample in IPA. After making the contacts, we wire‐bonded the contacts into a 12 pin sample holder. The Pd layer provided strength to the contacts, reducing the chance of gate breakdown during wire bonding.

### 4.3 AFM and PFM Imaging

We used an AIST‐NT SmartSPM to perform AFM and PFM. All AFM measurements were performed in tapping mode. In PFM, we used a platinum coated probe (Tap150E‐G) and applied an AC bias to the sample at the contact resonance frequency of the cantilever. The sample surface deflected in response to the AC bias as a result of the piezoelectric effect. A lock‐in amplifier captured the amplitude and phase of the cantilever deflection, where the amplitude was related to the magnitude of the piezoelectric moment and the phase was related to the ferroelectric orientation. We used PFM‐Top mode, where the probe contacts the sample intermittently for the PFM measurements but was lifted between two subsequent scanning points. The force was limited to less than 25 nN.

### 4.4 Electrical and Magnetotransport Measurements

We performed electrical transport at room‐temperature in a custom probe station under ambient conditions and the temperature‐dependent electrical and magnetotransport measurements in a Physical Property Measurement System (PPMS DynaCool from Quantum Design). We measured the output and transfer characteristics of the FETs using a Keithley 4200A‐SCS Parameter Analyzer. The source and drain electrode configurations for all FETs were indicated in Figures S1, S5, and S6. All measurements except for the magnetotransport were performed in a two‐point geometry. We performed the magnetotransport measurements in a four‐point device geometry, where all electrodes were aligned along the same edge of the CDW‐FET. For magnetotransport measurement, we used a SR830 lock‐in amplifier operated at a 13 Hz frequency and a 10 nA current bias amplitude and measured the voltage drop across the channel.

### 4.5 STEM Sample Preparation and Imaging

We prepared STEM samples for cross sectioning by taking already fabricated devices and applying a protective coating of amorphous carbon via thermal evaporation. We used a Thermo Fisher Scientific Helios 600i DualBeam FIB‐SEM to lift out and thin each TEM lamella, equipped with a cryo‐can during the thinning process. We used Thermo Fisher Scientific Themis Z aberration‐corrected STEM operating at 300 kV with a convergence angle of 25.2 mrad for STEM imaging.

## Author Contributions

S.M.N., A.M.v.d.Z., and P.Y.H. conceived the idea. Under supervision of A.M.v.d.Z. and S.W.N., S.M.N. fabricated the heterostructures and performed AFM and PFM characterization. Under supervision of A.M.v.d.Z. and N.M., S.M.N., and H.D. fabricated the FETs and performed the electrical and magnetotransport. S.M.N. analyzed the data. Under supervision of P.Y.H., G.N. performed the STEM imaging. S.M.N. and A.M.v.d.Z. wrote the manuscript. All authors read and contributed to the manuscript.

## Conflicts of Interest

The authors declare no conflicts of interest.

## Supporting information

Supporting Information

## Data Availability

The data that support the findings of this study are openly available in the Illinois Data Bank at https://doi.org/10.13012/B2IDB‐4015036_V2.

## References

[1] D. Meier and S. M. Selbach , “Ferroelectric Domain Walls for Nanotechnology,” Nature Reviews Materials 7, no. 3 (2022): 157–173.

[2] P. Sharma , T. S. Moise , L. Colombo , and J. Seidel , “Roadmap for Ferroelectric Domain Wall Nanoelectronics,” Advanced Functional Materials 32, no. 10 (2022): 2110263.

[3] G. F. Nataf , M. Guennou , J. M. Gregg , et al., “Domain‐Wall Engineering and Topological Defects in Ferroelectric and Ferroelastic Materials,” Nature Reviews Physics 2, no. 11 (2020): 634–648.

[4] P. S. Bednyakov , B. I. Sturman , T. Sluka , A. K. Tagantsev , and P. V. Yudin , “Physics and Applications of Charged Domain Walls,” npj Computational Materials 4, no. 1 (2018): 65.

[5] J. Seidel , L. W. Martin , Q. He , et al., “Conduction at Domain Walls in Oxide Multiferroics,” Nature Materials 8, no. 3 (2009): 229–234. 19169247 10.1038/nmat2373

[6] S. Van Aert , S. Turner , R. Delville , D. Schryvers , G. Van Tendeloo , and E. K. H. Salje , “Direct Observation of Ferrielectricity at Ferroelastic Domain Boundaries in ${\mathrm{CaTiO}}_{3}$ by Electron Microscopy,” Advanced Materials 24, no. 4 (2012): 523–527. 22223264 10.1002/adma.201103717

[7] Y. Geng , N. Lee , Y. J. Choi , S.‐W. Cheong , and W. Wu , “Collective Magnetism at Multiferroic Vortex Domain Walls,” Nano Letters 12, no. 12 (2012): 6055–6059. 23151028 10.1021/nl301432z

[8] M. Giraldo , Q. N. Meier , A. Bortis , et al., “Magnetoelectric Coupling of Domains, Domain Walls, and Vortices in a Multiferroic with Independent Magnetic and Electric Order,” Nature Communications 12, no. 1 (2021): 3093. 10.1038/s41467-021-22587-1 PMC8149668 34035244

[9] T. Sluka , A. K. Tagantsev , D. Damjanovic , M. Gureev , and N. Setter , “Enhanced Electromechanical Response of Ferroelectrics Due to Charged Domain Walls,” Nature Communications 3, no. 1 (2012): 748. 10.1038/ncomms1751 PMC4354168 22434191

[10] C. Stefani , L. Ponet , K. Shapovalov , et al., “Mechanical Softness of Ferroelectric $180^{\circ }$ Domain Walls,” Physical Review X 10 (2020): 041001.

[11] B. M. Vul , G. M. Guro , and I. I. Ivanchik , “Encountering Domains in Ferroelectrics,” Ferroelectrics 6, no. 1 (1973): 29–31.

[12] T. Sluka , A. K. Tagantsev , P. Bednyakov , and N. Setter , “Free‐Electron Gas at Charged Domain Walls in Insulating ${\mathrm{BaTiO}}_{3}$ ,” Nature Communications 4, no. 1 (2013): 1808. 10.1038/ncomms2839 PMC3674246 23651996

[13] H. Beccard , B. Kirbus , E. Beyreuther , et al., “Nanoscale Conductive Sheets in Ferroelectric ${\mathrm{BaTiO}}_{3}:$ Large Hall Electron Mobilities at Head‐to‐Head Domain Walls,” ACS Applied Nano Materials 5, no. 7 (2022): 8717–8722.

[14] C. S. Werner , S. J. Herr , K. Buse , et al., “Large and Accessible Conductivity of Charged Domain Walls in Lithium Niobate,” Scientific Reports 7, no. 1 (2017): 9862. 28851946 10.1038/s41598-017-09703-2 PMC5575345

[15] Z. Liu , H. Wang , M. Li , et al., “In‐Plane Charged Domain Walls with Memristive Behaviour in a Ferroelectric Film,” Nature 613, no. 7945 (2023): 656–661. 36653455 10.1038/s41586-022-05503-5

[16] J. A. Mundy , J. Schaab , Y. Kumagai , et al., “Functional Electronic Inversion Layers at Ferroelectric Domain Walls,” Nature Materials 16, no. 6 (2017): 622–627. 28319611 10.1038/nmat4878

[17] A. Q. Jiang , W. P. Geng , P. Lv , et al., “Ferroelectric Domain Wall Memory with Embedded Selector Realized in ${\mathrm{LiNbO}}_{3}$ Single Crystals Integrated on Si Wafers,” Nature Materials 19, no. 11 (2020): 1188–1194. 32541933 10.1038/s41563-020-0702-z

[18] D. Zhang , P. Schoenherr , P. Sharma , and J. Seidel , “Ferroelectric Order in van der Waals Layered Materials,” Nature Reviews Materials 8, no. 1 (2023): 25–40.

[19] W. Li , H. Liu , and H. Zeng , “Electric Field‐Controlled Interfacial Polarization Coupling in van der Waals Ferroelectric Heterojunctions,” Chinese Physics Letters 42, no. 5 (2025): 057501.

[20] F. Xue , W. Hu , K.‐C. Lee , et al., “Room‐Temperature Ferroelectricity in Hexagonally Layered α‐${\mathrm{In}}_{2}{\mathrm{Se}}_{3}$ Nanoflakes Down to the Monolayer Limit,” Advanced Functional Materials 28, no. 50 (2018): 1803738.

[21] J. A. Brehm , S. M. Neumayer , L. Tao , et al., “Tunable Quadruple‐Well Ferroelectric van der Waals Crystals,” Nature Materials 19, no. 1 (2020): 43–48. 31740791 10.1038/s41563-019-0532-z

[22] Z. Fei , W. Zhao , T. A. Palomaki , et al., “Ferroelectric Switching of a Two‐Dimensional Metal,” Nature 560, no. 7718 (2018): 336–339. 30038286 10.1038/s41586-018-0336-3

[23] E. Han , S. M. Nahid , T. Rakib , et al., “Bend‐Induced Ferroelectric Domain Walls in α‐${\mathrm{In}}_{2}{\mathrm{Se}}_{3}$ ,” ACS Nano 17, no. 8 (2023): 7881–7888. 37057994 10.1021/acsnano.3c01311

[24] S. M. Nahid , S. Nam , and A. M. van der Zande , “Depolarization Field‐Induced Photovoltaic Effect in Graphene/α‐${\mathrm{In}}_{2}{\mathrm{Se}}_{3}$/Graphene Heterostructures,” ACS Nano 18, no. 22 (2024): 14198–14206. 38771928 10.1021/acsnano.3c11558

[25] K. Yasuda , X. Wang , K. Watanabe , T. Taniguchi , and P. Jarillo‐Herrero , “Stacking‐Engineered Ferroelectricity in Bilayer Boron Nitride,” Science 372, no. 6549 (2021): 1458–1462. 10.1126/science.abd3230 34045323

[26] S. Farokhipoor and B. Noheda , “Conduction through 71 Domain Walls in ${\mathrm{BiFeO}}_{3}$ Thin Films,” Physical Review Letters 107 (2011): 127601. 22026801 10.1103/PhysRevLett.107.127601

[27] W. Wu , Y. Horibe , N. Lee , S.‐W. Cheong , and J. R. Guest , “Conduction of Topologically Protected Charged Ferroelectric Domain Walls,” Physical Review Letters 108 (2012): 077203. 22401247 10.1103/PhysRevLett.108.077203

[28] T. Choi , Y. Horibe , H. T. Yi , Y. J. Choi , W. Wu , and S.‐W. Cheong , “Insulating Interlocked Ferroelectric and Structural Antiphase Domain Walls in Multiferroic ${\mathrm{YMnO}}_{3}$ ,” Nature Materials 9, no. 3 (2010): 253–258. 20154694 10.1038/nmat2632

[29] R. G. McQuaid , M. P. Campbell , R. W. Whatmore , A. Kumar , and J. M. Gregg , “Injection and Controlled Motion of Conducting Domain Walls in Improper Ferroelectric Cu‐Cl Boracite,” Nature Communications 8, no. 1 (2017): 15105. 10.1038/ncomms15105 PMC5440803 28508870

[30] Y. S. Oh , X. Luo , F.‐T. Huang , Y. Wang , and S.‐W. Cheong , “Experimental Demonstration of Hybrid Improper Ferroelectricity and the Presence of Abundant Charged Walls in (Ca,Sr) Crystals,” Nature Materials 14, no. 4 (2015): 407–413. 25581628 10.1038/nmat4168

[31] I. Stolichnov , L. Feigl , L. J. McGilly , et al., “Bent Ferroelectric Domain Walls as Reconfigurable Metallic‐Like Channels,” Nano Letters 15, no. 12 (2015): 8049–8055. 26555142 10.1021/acs.nanolett.5b03450

[32] F. Risch , Y. Tikhonov , I. Lukyanchuk , A. M. Ionescu , and I. Stolichnov , “Giant Switchable Non Thermally‐Activated Conduction in 180 Domain Walls in Tetragonal Pb(Zr,Ti)$O_{3}$ ,” Nature Communications 13, no. 1 (2022): 7239. 10.1038/s41467-022-34777-6 PMC9700693 36433950

[33] G. Lindgren and C. Canalias , “Domain Wall Conductivity in ${\mathrm{KTiOPO}}_{4}$ Crystals,” APL Materials 5, no. 7 (2017): 076108.

[34] Y. Zhang , H. Lu , X. Yan , et al., “Intrinsic Conductance of Domain Walls in ${\mathrm{BiFeO}}_{3}$ ,” Advanced Materials 31, no. 36 (2019): 1902099. 10.1002/adma.201902099 31353633

[35] P. Maksymovych , J. Seidel , Y. H. Chu , et al., “Dynamic Conductivity of Ferroelectric Domain Walls in ${\mathrm{BiFeO}}_{3}$ ,” Nano Letters 11, no. 5 (2011): 1906–1912. 21486089 10.1021/nl104363x

[36] L. Liu , K. Xu , Q. Li , J. Daniels , et al., “Giant Domain Wall Conductivity in Self‐Assembled ${\mathrm{BiFeO}}_{3}$ Nanocrystals,” Advanced Functional Materials 31, no. 1 (2021): 2005876.

[37] M. S. Hossain , H. Lu , R. Khurana , et al., “Conducting Domain Walls in van der Waals Ferroelectric ${\mathrm{NbOI}}_{2}$ ,” Nano Letters 25, no. 37 (2025): 13844–13849. 40920544 10.1021/acs.nanolett.5c03386

[38] D. Meier , J. Seidel , A. Cano , et al., “Anisotropic Conductance at Improper Ferroelectric Domain Walls,” Nature Materials 11, no. 4 (2012): 284–288. 22367003 10.1038/nmat3249

[39] H. Lu , Y. Tan , J. P. V. McConville , et al., “Electrical Tunability of Domain Wall Conductivity in ${\mathrm{LiNbO}}_{3}$ Thin Films,” Advanced Materials 31, no. 48 (2019): 1902890. 10.1002/adma.201902890 31588637

[40] X.‐K. Wei , T. Sluka , B. Fraygola , et al., “Controlled Charging of Ferroelastic Domain Walls in Oxide Ferroelectrics,” ACS Applied Materials & Interfaces 9, no. 7 (2017): 6539–6546. 28141926 10.1021/acsami.6b13821

[41] M. Küpers , P. M. Konze , A. Meledin , et al., “Controlled Crystal Growth of Indium Selenide, ${\mathrm{In}}_{2}{\mathrm{Se}}_{3}$, and the Crystal Structures of α‐${\mathrm{In}}_{2}{\mathrm{Se}}_{3}$ ,” Inorganic Chemistry 57, no. 18 (2018): 11775–11781. 30153016 10.1021/acs.inorgchem.8b01950

[42] M. S. Choi , N. Ali , T. D. Ngo , et al., “Recent Progress in 1D Contacts for 2D‐Material‐Based Devices,” Advanced Materials 34, no. 39 (2022): 2202408. 10.1002/adma.202202408 35594170

[43] M. Si , A. K. Saha , S. Gao , et al., “A Ferroelectric Semiconductor Field‐Effect Transistor,” Nature Electronics 2, no. 12 (2019): 580–586.

[44] S. Chen , Y. Ning , C. S. Tang , et al., “ ${\mathrm{LaAlO}}_{3}$/${\mathrm{SrTiO}}_{3}$ Heterointerface: 20 Years and Beyond,” Advanced Electronic Materials 10, no. 3 (2024): 2300730.

[45] R. Chaudhuri , S. J. Bader , Z. Chen , D. A. Muller , H. G. Xing , and D. Jena , “A Polarization‐Induced 2D Hole Gas in Undoped Gallium Nitride Quantum Wells,” Science 365, no. 6460 (2019): 1454–1457. 31604274 10.1126/science.aau8623

[46] F. Lyu , Y. Sun , Q. Yang , et al., “Thickness‐Dependent Band Gap of α‐${\mathrm{In}}_{2}{\mathrm{Se}}_{3}:$ From Electron Energy Loss Spectroscopy to Density Functional Theory Calculations,” Nanotechnology 31, no. 31 (2020): 315711. 32294630 10.1088/1361-6528/ab8998

[47] H. Lee , N. Campbell , J. Lee , et al., “Direct Observation of a Two‐Dimensional Hole Gas at Oxide Interfaces,” Nature Materials 17, no. 3 (2018): 231–236. 29403056 10.1038/s41563-017-0002-4

[48] C.‐L. Jia , S.‐B. Mi , K. Urban , I. Vrejoiu , M. Alexe , and D. Hesse , “Atomic‐Scale Study of Electric Dipoles near Charged and Uncharged Domain Walls in Ferroelectric Films,” Nature Materials 7, no. 1 (2008): 57–61. 18066068 10.1038/nmat2080

[49] J. Gonnissen , D. Batuk , G. F. Nataf , et al., “Direct Observation of Ferroelectric Domain Walls in ${\mathrm{LiNbO}}_{3}:$ Wall‐Meanders, Kinks, and Local Electric Charges,” Advanced Functional Materials 26, no. 42 (2016): 7599–7604.

[50] G. Nolan , E. Han , S. M. Nahid , et al., “Atomic and Electronic Structure of Strongly Charged Domain Walls in van der Waals α‐${\mathrm{In}}_{2}{\mathrm{Se}}_{3}$ ,” arXiv:2601.19137 [cond‐mat.mtrl‐sci] (2026). 10.1021/acs.nanolett.6c00418 42007883

[51] Y. Wu , T. Zhang , D. Guo , et al., “Stacking‐Selected Polarization Switching and Phase Transition in VdW Ferroelectric α‐${\mathrm{In}}_{2}{\mathrm{Se}}_{3}$ Junction Devices,” Nature Communications 15, no. 1 (2024): 10481. 10.1038/s41467-024-54841-7 PMC11612147 39622832

[52] E. Han , J. Yu , E. Annevelink , et al., “Ultrasoft Slip‐Mediated Bending in Few‐Layer Graphene,” Nature Materials 19, no. 3 (2020): 305–309. 31712745 10.1038/s41563-019-0529-7

[53] X. Cui , G.‐H. Lee , Y. D. Kim , et al., “Multi‐Terminal Transport Measurements of ${\mathrm{MoS}}_{2}$ Using a van der Waals Heterostructure Device Platform,” Nature Nanotechnology 10, no. 6 (2015): 534–540. 10.1038/nnano.2015.70 25915194

[54] Y. Park , H. W. Baac , J. Heo , and G. Yoo , “Thermally Activated Trap Charges Responsible for Hysteresis in Multilayer ${\mathrm{MoS}}_{2}$ Field‐Effect Transistors,” Applied Physics Letters 108, no. 8 (2016): 083102.

[55] D. Jariwala , V. K. Sangwan , D. J. Late , et al., “Band‐Like Transport in High‐Mobility Unencapsulated Single‐Layer ${\mathrm{MoS}}_{2}$ Transistors,” Applied Physics Letters 102, no. 17 (2013): 173107.

[56] N. Mott , “Conduction in Glasses Containing Transition Metal Ions,” Journal of Non‐Crystalline Solids 1, no. 1 (1968): 1–17.

[57] N. F. Mott , M. Pepper , S. Pollitt , R. H. Wallis , and C. J. Adkins , “The Anderson Transition,” Proceedings of the Royal Society of London A, Mathematical and Physical Sciences 345, no. 1641 (1975): 169–205.

[58] G. Bergmann , “Weak Localization in Thin Films: A Time‐of‐Flight Experiment with Conduction Electrons,” Physics Reports 107, no. 1 (1984): 1–58.

[59] M. A. Khan , J. N. Kuznia , J. M. Van Hove , N. Pan , and J. Carter , “Observation of a Two‐Dimensional Electron Gas in Low‐Pressure Metalorganic Chemical Vapor Deposited GaN‐${\mathrm{Al}}_{x}{\mathrm{Ga}}_{1-x}N$ Heterojunctions,” Applied Physics Letters 60, no. 24 (1992): 3027–3029.

[60] H. Tampo , H. Shibata , K. Maejima , et al., “Polarization‐Induced Two‐Dimensional Electron Gases in ZnMgO/ZnO Heterostructures,” Applied Physics Letters 93, no. 20 (2008): 202104.

[61] C. Noguera , “Polar Oxide Surfaces,” Journal of Physics: Condensed Matter 12, no. 31 (2000): R367.

[62] A. Castellanos‐Gomez , M. Buscema , R. Molenaar , et al., “Deterministic Transfer of Two‐Dimensional Materials by All‐Dry Viscoelastic Stamping,” 2D Materials 1, no. 1 (2014): 011002.

