# Supplementary material for: Field‐Effect Transistors from Artificial Charged Domain Walls in Stacked Van der Waals Ferroelectric α‐In2Se3
Source: Adv Mater. 2026 Jan 30;38(20):e23096. doi: 10.1002/adma.202523096 (PMC13054130; doi:10.1002/adma.202523096)
Supplement: Supplementary file 1 — Supporting Information [file ADMA-38-e23096-s001.pdf]

# Supporting Information for Field-Effect Transistors from Artificial Charged Domain Walls in Stacked van der Waals Ferroelectric $\alpha$-$In_2Se_3$

*Shahriar Muhammad Nahid Haiyue Dong Gillian M. Nolan SungWoo Nam Nadya Mason Pinshane Y. Huang Arend M. van der Zande**

Shahriar Muhammad Nahid
Department of Mechanical Science and Engineering; Grainger College of Engineering
University of Illinois Urbana–Champaign, Urbana, IL 61801, USA
Email: snahid2@illinois.edu

Haiyue Dong
Department of Physics; Grainger College of Engineering
University of Illinois Urbana–Champaign, Urbana, IL 61801, USA
Email: haiyued2@illinois.edu

Gillian Nolan
Department of Materials Science and Engineering; Grainger College of Engineering
University of Illinois Urbana–Champaign, Urbana, IL 61801, USA
Email: gnolan2@illinois.edu

SungWoo Nam
Department of Mechanical and Aerospace Engineering; Department of Materials Science and Engineering
University of California, Irvine, Irvine, CA 92697, USA
Email: sungwoo.nam@uci.edu

Nadya Mason
Pritzker School of Molecular Engineering
University of Chicago, Chicago, IL 60637, USA
Email: nmason1@uchicago.edu

Pinshane Y. Huang
Department of Materials Science and Engineering; Materials Research Laboratory; Grainger College of Engineering
University of Illinois Urbana–Champaign, Urbana, IL 61801, USA
Email: pyhuang@illinois.edu

Arend M. van der Zande*
Department of Mechanical Science and Engineering; Department of Materials Science and Engineering; Materials Research Laboratory; Grainger College of Engineering
University of Illinois Urbana–Champaign, Urbana, IL 61801, USA
Email: arendv@illinois.edu

# 1 Discussion of ferroelectric heterostructure types and yield via aligned transfer

Four different kinds of heterostructures can be formed via van der Waals stacking of out-of-plane ferroelectrics: H-H (pointing towards each other), H-T (both pointing up), T-H (both pointing down), and T-T (both pointing away from each other). These combinations ignore how interlayer twist might interact with out-of-plane dipole moments. In our procedure, we create the heterostructures inside a glove box using exfoliation and aligned transfer techniques, and only characterize the polarization by piezoelectric force microscopy (PFM) phase after fabrication. This process allow us to minimize the chance of oxidization or physisorption of hydrocarbons on the charged ferroelectric surface before construction of the heterostructures. Because the exfoliation process is entirely random, a simple hypothesis predicts an equal probability of 25% to create each of the four structures. However, we find that the yield is not equally distributed. Of 41 heterostructures created, we find that 15 are H-H domain walls (37%), 12 are H-T (29%), 13 are T-H (32%), and only 1 form a T-T (2%). In other words, the yield of the T-T domain walls is surprisingly low and statistically significant ($p = 0.0002$, one-sided binomial test). There are two possibilities: either there is a systematic error in how the domain walls are fabricated or there is a difference in the energetic stability of the T-T domain wall. We note that the yield of the H–H domain walls (15 out of 41, or 37%) is higher than the 25 % expected from our simple hypothesis of equal probability. However, this deviation is not statistically significant ($p = 0.066$, one-sided binomial test). Furthermore, in a separate study,[1] we explore the structure of naturally-occurring domain walls and find that the T-T domain wall has different structure and a lower barrier energy to migration from the H-H domain wall. Thus, we hypothesize that the T-T domain walls are less stable than the H-H ones and thus flip into a H-T or T-H heterostructure during the elevated temperatures used in the aligned transfer process. As a consequence, although we have tried for a significant period to fabricate a T-T CDW FET for additional comparison in this study, we were unable to produce a functioning device due to low yield.

# 2 Impact of device geometry on the CDW conductivity comparison

The conductance comparison in Figure 1f between the in-plane CDW-FETs reported in this work and previously reported out-of-plane CDWs involves different device geometries. The CDW-FETs utilize lithographically defined edge contacts, whereas most previous studies relied on conductive atomic force microscopy (CAFM) based techniques, which injects current through nanoscale point contact with an unspecified contact area determined by the contact mechanics and an ill-defined current path through the film thickness. In addition, many previous studies did not report key experimental parameters, such as probe diameter or channel length, further complicating the conductivity comparison. Furthermore, the inherent 2D nature of the CDWs adds further ambiguity in defining the channel thickness. As a result, direct comparison of 3D conductivity remains challenging, and we therefore report 2D conductivity, which is independent of channel thickness.

Nevertheless, in Table S2, we evaluate the 2D conductivity using conservative estimates for the geometric factors. For example, we used the probe diameter as the contact width instead of the contact mechanics defined diameter and considered the thickness as the channel length. Even with these conservative estimates, Table S2 shows that the 2D conductivity of H-H #3 exceeds the best reported out-of-plane CDWs by more than two orders of magnitude, confirming that the observed superior performance is not an artifact of geometric bias.

# 3 Polar discontinuity as the origin of enhanced CDW conductance

In the manuscript, we attribute enhanced conductance in CDW-FETs to polarization bound charge induced interfacial band bending and subsequent carrier screening. However, in heterointerfaces, enhanced conductance can also arise from non-polar mechanisms that are independent of polarization, such as local stoichiometric variations or defect metallization. In this section, we critically examine these non-polar

mechanisms and demonstrate that they do not account for the enhanced conductance observed in CDW-FETs.

Stoichiometric variations can lead to enhanced conductance by introducing excess carriers or metallic secondary phases at an interface. However, none of the known phases of $In_2Se_3$, including the $\beta$-$In_2Se_3$ and amorphous phase, is metallic in bulk.[2, 3] In addition, unlike epitaxially grown interface, where lattice matching and dangling bonds create stoichiometric variations, van der Waals heterostructures preserve the stoichiometry of the constituent layers.[4] Therefore, stoichiometric variations alone, without the influence of polarization bound charges, do not account for the enhanced conductance in CDW-FETs.

Another possible non-polar origin of enhanced conductance is metallization of defects generated from vacancies or oxidization during transfer. We exclude this mechanism as the dominant contributor to CDW-FET conductance for three reasons. First, the hysteresis in the H-T FET indicates the presence of similar interfacial defects as in H-H FETs. However, the H-T FET does not exhibit enhanced conductance. Second, the low temperature transport in CDW-FETs follows variable range hopping (VRH), indicating that charge transport occurs via hopping between localized states with 10s of nm length scales, rather than through delocalized impurity bands with sub-nm length scales. Third, the hysteresis observed at all temperatures is consistent with charge trapping and de-trapping mechanisms, rather than metallic conduction from defects.

Together, these observations reinforce polarization-induced band bending as the origin of enhanced CDW conductance.

## 4 Origin of hysteresis in the CDW-FET transfer curves

The hysteresis in CDW-FET originates from ferroelectric switching[5] or charge trapping.[6] We rule out ferroelectric switching as the cause of hysteresis for three reasons. First, the gate dielectric (285 nm $SiO_2$) is much thicker than the ferroelectric domains. If we assume no bulk screening and consider the gate dielectric and bottom ferroelectric domain as series capacitors, the maximum applied gate bias-induced electric field across the ferroelectric domain is less than 0.03 V/nm for single domain, H-T, and H-H #1-2 FETs and 0.14 V/nm for H-H #3. These values are much smaller than the coercive field of $\alpha$-$In_2Se_3$ (0.33 V/nm).[7] Considering bulk screening, the actual electric field value is likely even smaller. Second, hysteresis due to ferroelectric switching should consistently appear in the transfer curves of single domain, H-T, and H-H CDW-FET at all temperatures. However, the single domain FET does not exhibit significant hysteresis at low temperatures. Finally, the polarization of each domain in the H-H CDW-FET is consistent between the PFM, conducted before transport measurement, and STEM imaging, performed afterward, confirming the absence of polarization switching. For these reasons, we exclude macroscopic polarization switching as the origin of hysteresis in the FETs.

We also note that, although macroscopic polarization switching is excluded, microscopic polarization effects can occur, such as defect dipole reorientation, localized domain wall motion, or heterogeneous phase changes at the metastable interface. These effects are expected to be localized. The absence of any abrupt change in transfer curves and their consistent behavior under repeated sweeps indicate that such effects play only a minor role in transport. Accordingly, we confirm charge trapping as the primary origin of hysteresis.

## 5 Effects of CDW heterogeneity on local band structure

The polar discontinuity at the CDW induces band bending at the interface. However, structural heterogeneity in these artificial CDWs, as illustrated in Figure 2 and Figure S7, causes variations in the magnitude of the band bending and introduces trap states. In this section, we discuss how such heterogeneity governs the local band structures and associated trap states. We focus on the three representative atomic configurations depicted in Figure 2: $\beta$-$In_2Se_3$, amorphous regions, and nanogaps.

1. In a separate study, we have shown that naturally occurring H-H CDWs in $\alpha$-$In_2Se_3$ contain a monolayer of$\beta$-$In_2Se_3$. The bulk $\beta$-$In_2Se_3$ is also a semiconductor, with a different chemical potential than $\alpha$-

$In_2Se_3$. DFT calculations show that when $\beta$-$In_2Se_3$ is sandwiched between two opposite polar domains of $\alpha$-$In_2Se_3$ in a H-H CDW, the heterostructure shifts the $\beta$-$In_2Se_3$ bands down, introducing highly confined midgap bands in the bulk bandgap.[1]

2. The amorphous layer is of similar thickness and should correspond to similar stoichiometry as monolayer $In_2Se_3$, albeit with reduced crystallinity. Thus, along the z direction, both the $\beta$-$In_2Se_3$ and amorphous regions lead to an abrupt band bending at the CDW, though the magnitude will be different (Figure S8a).

3. In the nanogap regions, the top and bottom domains are the farthest from each other. At these larger separations, the effect of opposite polar domains weakens and results in smaller band bending (Figure S8b).

Determining the origins of trap states in any system is challenging, as there can be many contributing factors. For example, they could rise from the nanoscale in-plane confinement of the different phases, bulk defects that get tuned to a different energy at the domain wall, boundaries between different phases, or atomic vacancies or oxidization created during fabrication. Together, these distinct band diagrams create an in-plane spatial variation in the bound charge density, band edges, and trap density, depicted in Figure 3d. However, a quantitative determination of the energetics associated with each structure is beyond the scope of this work.

# 6 Origin of device-to-device variability in CDW-FET performance

Several extrinsic factors give rise to the four orders of magnitude device-to-device variation observed in Figure 1f. As discussed in section 5 and displayed in Figure S8, nanogaps exhibit the weakest band bending, while structure containing $\beta$-$In_2Se_3$ and amorphous regions exhibit stronger band bending, accompanied by trap states. Consequently, devices with a higher fraction of $\beta$-$In_2Se_3$ and amorphous layer should exhibit higher conductance with increased hysteresis, while those with more nanogaps will show lower conductance and reduced hysteresis.

The exact atomic structure of the interface and consequently the device performance depends on several uncontrolled parameters, including surface roughness of each domain due to thickness variation, relative twist angle between the top and bottom domains, local strain introduced during transfer, trapped bubbles, and doping. Variation in these factors will modify the interfacial morphology and the CDW-FET conductance. For instance, increased thickness nonuniformity in the constituent domains generate a higher density of nanogaps at the interface, leading to reduced conductance in the corresponding CDW-FET. In addition, imperfect edge contacts enhance the contact resistance, further decreasing overall device conductance. Future studies should focus on systematically controlling these parameters to quantitatively relate structural variations with device performance.

In addition, imperfect edge contacts enhance the contact resistance, further decreasing overall device conductance. Collectively, these extrinsic factors give rise to the device-to-device variations in Figure 1f.

# 7 Models for transport in H-H CDW

We observe two distinct modes of conduction in $\alpha$-$In_2Se_3$ H-H CDW: Mott variable range hopping (VRH) at temperatures below 80 K and thermally activated trap-assisted transport above 80 K.

Mott VRH model predicts the following scaling of conductance ($G$) with temperature:[8, 9]

$$G = G_0 \exp[-(\frac{T_0}{T})^{1/3}] \tag{1}$$

Here, $G_0$ is the prefactor, $T$ is the temperature, $T_0 = \frac{13.8}{K_B N(E_F)\xi^2}$ is the characteristic temperature, $K_B$ is the Boltzmann constant, $N(E_F)$ is the density of states at Fermi energy $E_F$, and $\xi$ is the localization length. The hopping length $r_{hop}$ is related to $\xi$, $r_{hop} = \frac{1}{4}\xi(\frac{T_0}{T})^{\frac{1}{3}}$.[10] We estimate the $r_{hop}$ from the conductance versus temperature fitting in Figure 4a. $N(E_F)$ is extracted from the subthreshold swing.[11, 12]

At high temperature, thermally activated traps lead to hysteresis in the transfer curves and follow the Arrhenius relation:

$$\Delta n = n_{shallow} + n_{thermal} \exp(-\frac{E_a}{K_B T}) \tag{2}$$

Here, $\Delta n$ is the trap density, $n_{shallow}$ is the trap density of shallow states with activation energy much smaller than the thermal energy at measured temperatures, and $n_{thermal}$ is the maximum density of thermally activated traps, $E_a$ is the activation energy, and $K_B T$ is the thermal energy. The trap states are activated only when the thermal energy is sufficient compared to the activation energy of the traps. We calculate the trap density from the threshold voltage difference between up and down sweeps using the following equation:

$$\Delta n = \frac{C_{ox}(V_{th\_down} - V_{th\_up})}{q} \tag{3}$$

Here, $C_{ox}$ is the capacitance of the $SiO_2$ dielectric, and $q$ is the electron charge. For quantifying $C_{ox}$, we assume a parallel plate capacitor geometry with a $SiO_2$ dielectric constant of 3.9 and a thickness of 285 nm.

Similar to the conductance, field-effect mobility ($\mu$) also shows two regimes. Below 80 K, it follows the scaling of Mott VRH ($\mu_{VRH} \propto (\frac{T_0}{T})^{\frac{1}{3}}$). Above 80 K, scattering with traps limit the mobility ($\mu_{trap} \propto exp(\frac{E_a}{K_B T})$). We fit the mobility data according to Matthiessen's rule, $\frac{1}{\mu} = \frac{1}{\mu_{VRH}} + \frac{1}{\mu_{trap}}$.

The Hikami–Larkin–Nagaoka (HLN) model describes the weak localization observed in the magneto-transport measurement at 2 K, shown in Figure 4c. According to the model, the longitudinal conductance change $\Delta Gxx$ under a magnetic field $B$ is:

$$\Delta Gxx = \frac{\alpha e^2}{2\pi^2 \hbar}[\psi(\frac{1}{2} + \frac{B_\phi}{B}) - ln(\frac{B_\phi}{B})] \tag{4}$$

Here, $\alpha$ is an empirical fitting parameter, $\psi$ is the digamma function, $B_\phi = \frac{\hbar}{4eL_\phi^2}$ is the characteristic magnetic field, and $L_\phi$ is the phase coherence length.

# 8 Supporting Table and Figures

| **FET Device Label** | **Channel Length ($\mu$m)** | **Contact Width ($\mu$m)** | **Average Flake Thickness (nm)** |
|---|---|---|---|
| Single Domain | 2.5 | 4 | 32.0 |
| H-H#1 | 7 | 6 | 50.7 and 37.1 |
| H-H#2 | 20 | 6 | 23.4 and 67.1 |
| H-H#3 | 2 | 1 | 57.1 and 65.2 |
| H-T | 5 | 5.5 | 43.9 and 39.5 |

Table S1: Dimension of each FET measured in this study.

| **Material** | **Method** | **Channel** Length ($\mu$m) | **Contact** Width ($\mu$m) | **Conductance** ($1/\Omega$) | **2D Conductivity** ($1/\Omega$) | **Ref.** |
|---|---|---|---|---|---|---|
| $\alpha$-$In_2Se_3$, H-H #1 | CDW-FET | 7 | 6 | 4.0E-06 | 4.6E-06 | This study |
| $\alpha$-$In_2Se_3$, H-H #2 | CDW-FET | 20 | 6 | 2.4E-08 | 7.9E-08 | This study |
| $\alpha$-$In_2Se_3$, H-H #3 | CDW-FET | 2 | 1 | 3.2E-04 | 6.4E-04 | This study |
| $NbOI_2$ | CAFM | 0.1 | 0.06 | 3.2E-12 | 5.3E-12 | Hossain et al. [13] |
| BFO | CAFM | 0.1 | - | 2.5E-13 | - | Seidel et al. [14] |
| BFO | CAFM | 0.04-0.07 | - | 7.5E-13 | - | Farokhipoor et al. [15] |
| BFO | CAFM | 0.2-0.4 | - | 4.5E-12 | - | Zhang et al. [16] |
| BFO | CAFM | 0.1 | - | 1.0E-10 | - | Maksymovych et al. [17] |
| BFO | CAFM | 0.12 | 0.04 | 1.0E-06 | 3.0E-06 | Liu et al. [18] |
| BTO | Top Electrodes | 200 | 200 | 1.0E-08 | 1.0E-08 | Sluka et al. [19] |
| LNO | CAFM | 300 | 2 | 1.1E-08 | 1.7E-06 | Werner et al. [20] |
| LNO | Top Electrodes | 0.2 | 0.2 | 1.8E-06 | 1.8E-06 | Jiang et al. [21] |
| LNO | CAFM | 0.5 | 0.05 | 11.8E-12 | 11.8E-11 | Lu et al. [22] |
| EMO | CAFM | 1000 | 0.1 | 1.7E-11 | 1.7E-07 | Mundy et al. [23] |
| EMO | CAFM | 200 | - | 2.0E-11 | - | Meier et al. [24] |
| HMO | CAFM | - | - | 4.9E-11 | - | Wu et al. [25] |
| YMO | CAFM | 30 | 0.06 | 5.0E-11 | 2.5E-08 | Choi et al. [26] |
| $Cu_3B_7O_{13}Cl$ | CAFM | 500 | - | 9.2E-14 | - | McQuaid et al. [27] |
| $(Ca,Sr)_3Ti_2O_7$ | CAFM | - | - | 9.2E-10 | - | Oh et al [28] |
| PZT | CAFM | 0.06 | 0.1 | 3.9E-12 | 2.3E-12 | Stolichnov et al. [29] |
| PZT | CAFM | 0.06 | - | 5.0E-08 | - | Risch et al. [30] |
| PZT | CAFM | 0.12 | 0.05 | 5.3E-11 | 1.3E-10 | Wei et al. [31] |
| $KTiOPO_4$ | CAFM | 100 | 0.06 | 1.4E-12 | 2.4E-09 | Lindgren at al. [32] |

Table S2: Comparison of the conductance and 2D conductivity of different CDWs.

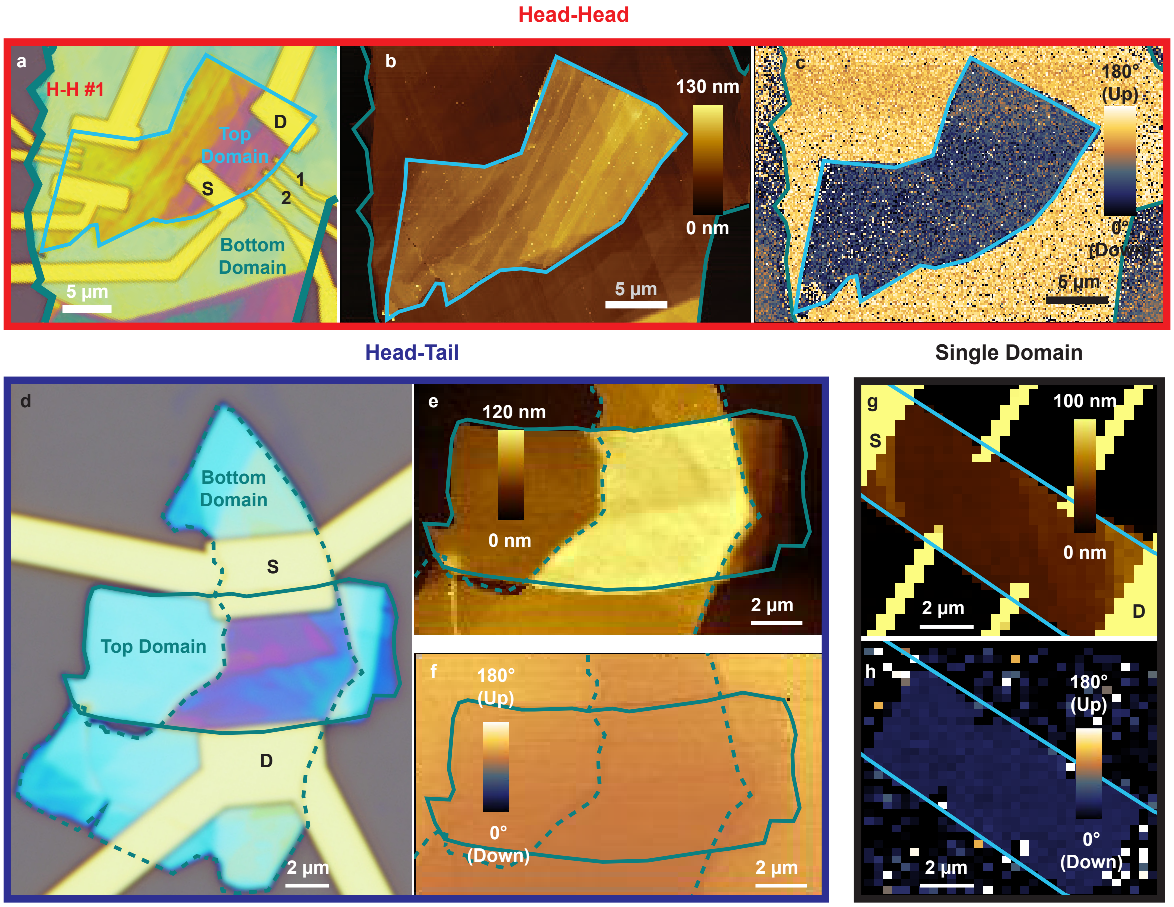


**Figure S1: Optical, atomic force microscopy (AFM), and piezoelectric force microscopy (PFM) images of (a-c), H-H #1,(d-f), H-T, and (g-h), single domain sample. a,** Optical microscope image of H-H #1. The cyan and teal colors denote the top (down polarization) and bottom domain (up polarization). The letters S and D denote the source and drain electrodes used for the FET characterization and numbers 1 and 2 denote the voltage probes for magnetotransport. The left side electrodes are excluded from measurements due to poor wire-bonding quality. **b,** AFM image showing the topography of the heterostructure. **c,** PFM confirms the polarization of each domain—$P_{down}$ in the top one and $P_{up}$ in the bottom one. **d,** Optical microscope image of H-T structure. Dashed teal line denotes the bottom domain whereas the solid teal line denotes the top domain. **e-f** AFM and PFM showing the topography and polarization (both domains have up polarization). **g-h** AFM and PFM image of single domain. Similar to (a), the letters S and D in d, and g denote the source and drain electrodes.

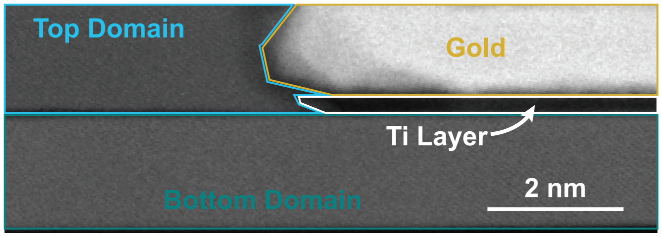


**Figure S2: STEM image of electrical contact with the CDW.** The top and bottom domains are marked in cyan and teal. Golden and white colored borders refer to the deposited gold and Ti layers. Ti touches the CDW through the edge.

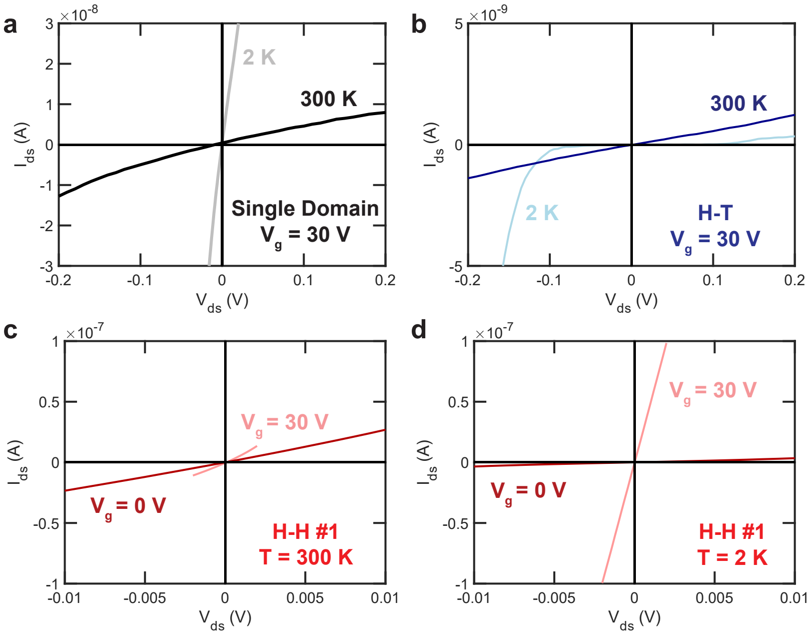


**Figure S3: Output curves of single domain $\alpha$-$In_2Se_3$, H-T, and H-H CDW-FET. a-b,** Output curves of (a) single domain $\alpha$-$In_2Se_3$ and (b) H-T FET at 2 K and 300 K. The output curves are measured at $V_g = 30$ V. **c-d,** Output curves of H-H CDW-FET, measured at $V_g = 0$ V and 30 V, at (c) 300 K and (d) 2 K. H-H CDW-FET curves are linear, confirming ohmic contact.

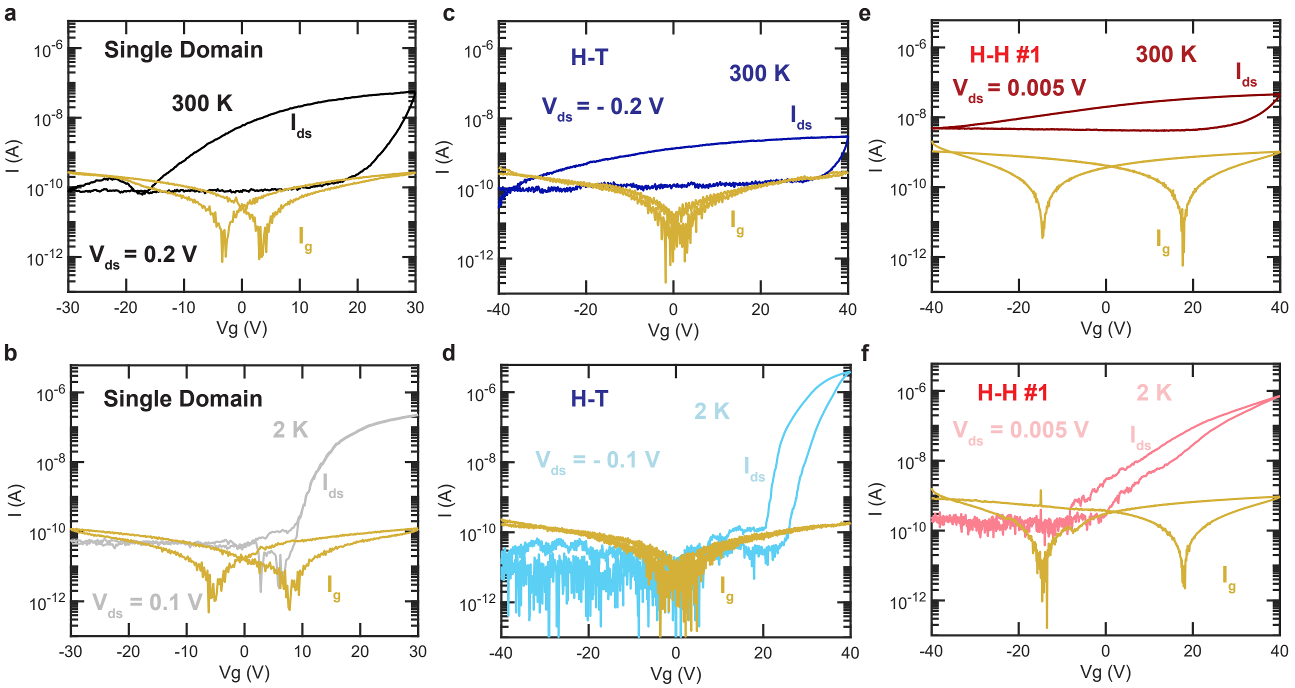


**Figure S4: Comparison of the transfer curves and gate leakage current ($I_g$) of single domain $\alpha$-$In_2Se_3$, H-T, and H-H #1.** $I_{ds}$ and $I_g$ versus gate voltage for **a-b,** single domain $\alpha$-$In_2Se_3$, **c-d,** H-T, and **e-f,** H-H #1 at 300 K and 2 K. Gate leakage is below 1 nA for all the cases.

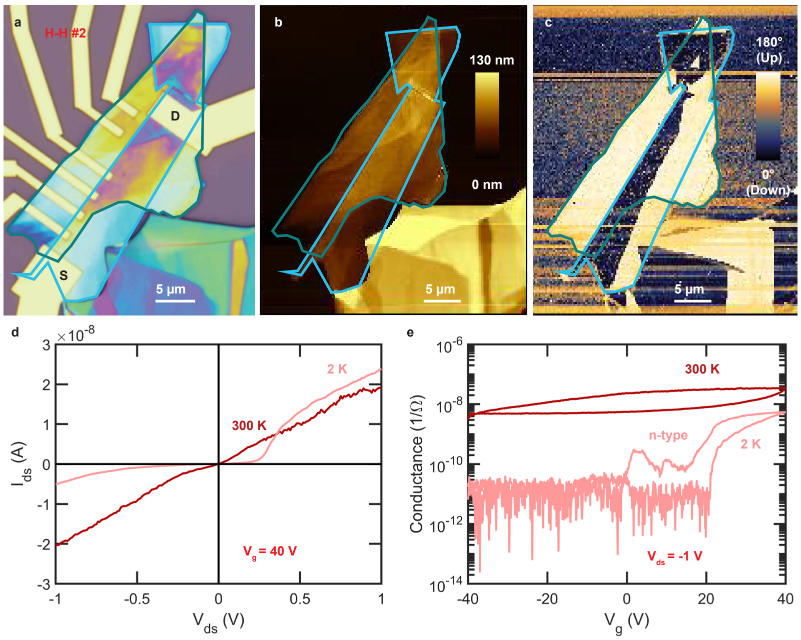


**Figure S5: AFM, PFM, and transport characterization of H-H #2. a-c,** Optical, AFM, PFM images of H-H #2. S and D denote the source and drain electrodes used for FET characterization. AFM and PFM are performed before putting the electrodes. Cyan and teal lines determine the top and bottom domains. PFM confirms the H-H configuration. **d-e,** Output curves ($V_g$ = 40 V) and transfer curve ($V_{ds}$ = -1 V) at 2 K and 300 K. Transfer curve shows 'always on' at 300 K, but transitions into n-type at 2 K.

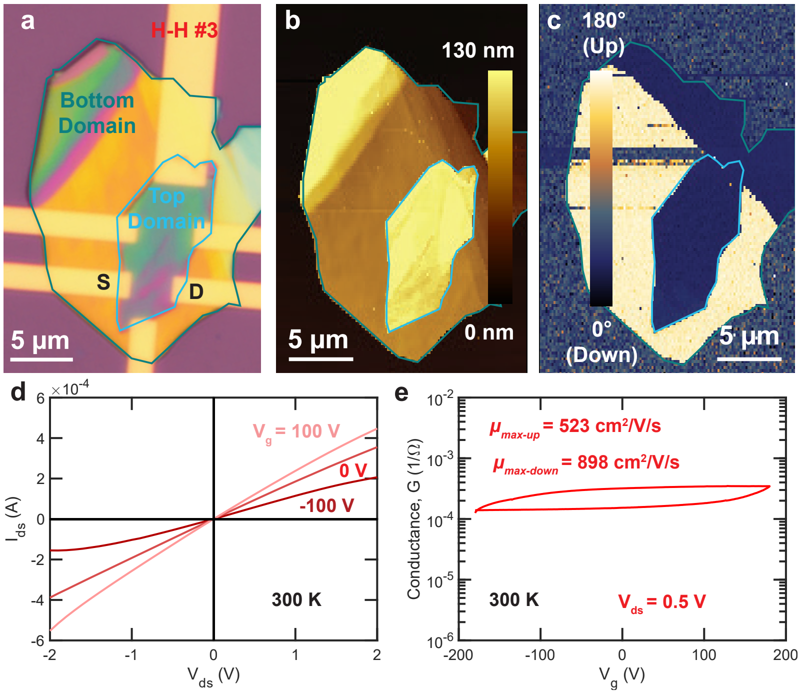


**Figure S6: Characterization and transport of H-H #3 a-c,** Optical, AFM, PFM images of H-H #3. S and D are the source and drain electrodes for FET measurement. AFM and PFM are performed before putting the electrodes. Cyan and teal lines determine the top and bottom flake. PFM confirms the H-H configuration. **d-e,** Output curves at $V_g$ = -100, 0, 100 V and transfer curve at $V_{ds}$ = 1 V at 300 K. Output curves show linear behavior, confirming ohmic contact. Transfer curve shows less than 1 order of magnitude of modulation under gating, similar to the one demonstrated for H-H #1 in the main manuscript. The maximum field-effect mobility is 523 and 898 $cm^2/V/s$ for up-sweep and down-sweep conditions.

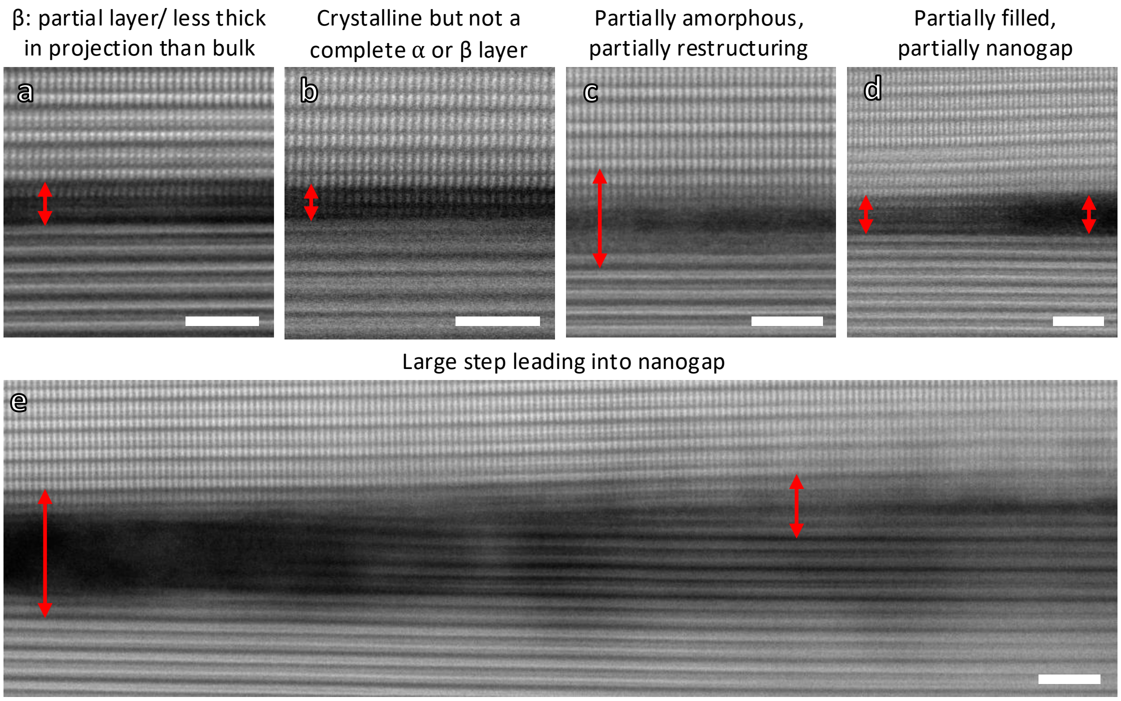


**Figure S7: ADF-STEM images displaying various features/structures found in the interface of H-H #1.** Interface containing **a,** $\beta$-$In_2Se_3$, **b,** region with atomic columns that do not correspond to a 5-layer $\alpha$-$In_2Se_3$ structure, **c,** regions of amorphized $\alpha$-$In_2Se_3$ alongside partial reconstruction of atomic structure, **d,** partially reconstructed $\alpha$-$In_2Se_3$ and partially nanogap. **e,** Wider field of view showing nanogap formed by a large step in bottom $\alpha$-$In_2Se_3$ flake. All scale bars are 2 nm. Red arrows denote the effective gap between flakes.

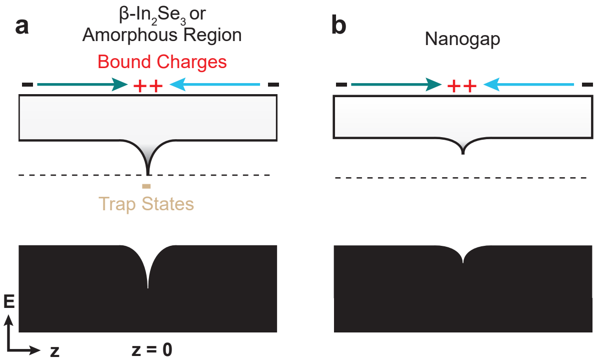


**Figure S8: Out-of-plane band diagram showing band bending at the H-H CDW for different interfacial atomic configurations.** Band diagram of **a**, $\beta$-$In_2Se_3$ or amorphous region and **b,** nanogap. Structures with $\beta$-$In_2Se_3$ layer and amorphous region lead to stronger band bending with trap states, while nanogap show weak band bending.

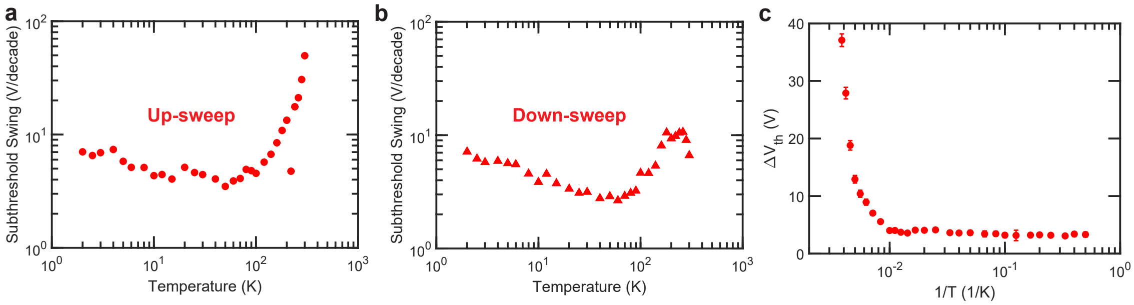


**Figure S9: Temperature scaling of the subthreshold swing and threshold voltage difference for CDW-FET (H-H #1). a-b,** Subthreshold swing versus temperature for up-sweep and down-sweep. **c,** Threshold voltage difference versus temperature.

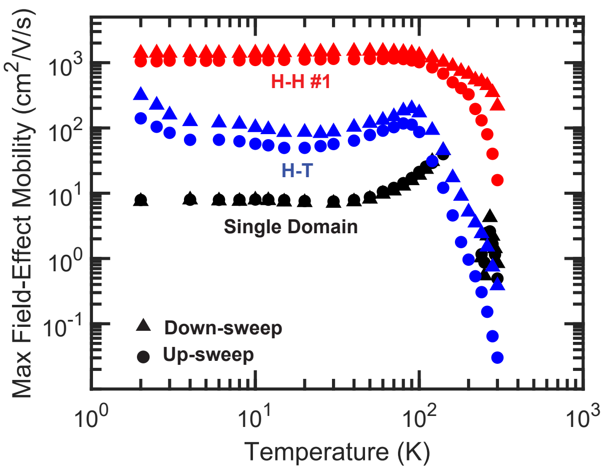


**Figure S10: Maximum field-effect mobility of single domain (black), H-T (blue), and H-H #1 (red) for both up-sweep (circles) and down-sweep (triangles).**

# References

[1] G. Nolan, E. Han, S. M. Nahid, P. Carmichael, A. van der Zande, A. Schleife, P. Huang, Atomic and electronic structure of strongly charged domain walls in van der waals $\alpha$-$In_2Se_3$, *arXiv:2601.19137 [cond-mat.mtrl-sci]* **2026**.

[2] M. Afifi, N. Hegab, A. Bekheet, Effect of annealing on the electrical properties of $In_2Se_3$ thin films, *Vacuum* **1995**, *46*, 4 335.

[3] H. T. El-Shair, A. E. Bekheet, Effect of heat treatment on the optical properties of $In_2Se_3$ thin films, *Journal of Physics D: Applied Physics* **1992**, *25*, 7 1122.

[4] Y. Liu, Y. Huang, X. Duan, Van der waals integration before and beyond two-dimensional materials, *Nature* **2019**, *567*, 7748 323.

[5] M. Si, A. K. Saha, S. Gao, G. Qiu, J. Qin, Y. Duan, J. Jian, C. Niu, H. Wang, W. Wu, S. K. Gupta, P. D. Ye, A ferroelectric semiconductor field-effect transistor, *Nature Electronics* **2019**, *2*, 12 580.

[6] Y. Park, H. W. Baac, J. Heo, G. Yoo, Thermally activated trap charges responsible for hysteresis in multilayer $MoS_2$ field-effect transistors, *Applied Physics Letters* **2016**, *108*, 8 083102.

[7] W. F. Io, S. Yuan, S. Y. Pang, L. W. Wong, J. Zhao, J. Hao, Temperature-and thickness-dependence of robust out-of-plane ferroelectricity in CVD grown ultrathin van der Waals $\alpha$-$In_2Se_3$ layers, *Nano research* **2020**, *13* 1897.

[8] N. Mott, Conduction in glasses containing transition metal ions, *Journal of Non-Crystalline Solids* **1968**, *1*, 1 1.

[9] N. F. Mott, M. Pepper, S. Pollitt, R. H. Wallis, C. J. Adkins, The Anderson transition, *Proceedings of the Royal Society of London. A. Mathematical and Physical Sciences* **1975**, *345*, 1641 169.

[10] S. I. Khondaker, I. S. Shlimak, J. T. Nicholls, M. Pepper, D. A. Ritchie, Two-dimensional hopping conductivity in a $\delta$-doped $GaAs/Al_xGa_{1-x}As$ heterostructure, *Physical Review B* **1999**, *59* 4580.

[11] D. Jariwala, V. K. Sangwan, D. J. Late, J. E. Johns, V. P. Dravid, T. J. Marks, L. J. Lauhon, M. C. Hersam, Band-like transport in high mobility unencapsulated single-layer $MoS_2$ transistors, *Applied Physics Letters* **2013**, *102*, 17 173107.

[12] M.-G. Kim, H. S. Kim, Y.-G. Ha, J. He, M. G. Kanatzidis, A. Facchetti, T. J. Marks, High-performance solution-processed amorphous zinc-indium-tin oxide thin-film transistors, *Journal of the American Chemical Society* **2010**, *132*, 30 10352.

[13] M. S. Hossain, H. Lu, R. Khurana, M. I. Kholil, S. Bagheri, J. Abourahma, A. Sinitskii, A. Gruverman, Conducting domain walls in van der waals ferroelectric $NbOI_2$, *Nano Letters* **2025**, *25*, 37 13844, pMID: 40920544.

[14] J. Seidel, L. W. Martin, Q. He, Q. Zhan, Y.-H. Chu, A. Rother, M. E. Hawkridge, P. Maksymovych, P. Yu, M. Gajek, N. Balke, S. V. Kalinin, S. Gemming, F. Wang, G. Catalan, J. F. Scott, N. A. Spaldin, J. Orenstein, R. Ramesh, Conduction at domain walls in oxide multiferroics, *Nature Materials* **2009**, *8*, 3 229.

[15] S. Farokhipoor, B. Noheda, Conduction through 71° domain walls in $BiFeO_3$ thin films, *Physical Review Letters* **2011**, *107* 127601.

[16] Y. Zhang, H. Lu, X. Yan, X. Cheng, L. Xie, T. Aoki, L. Li, C. Heikes, S. P. Lau, D. G. Schlom, L. Chen, A. Gruverman, X. Pan, Intrinsic conductance of domain walls in $BiFeO_3$, *Advanced Materials* **2019**, *31*, 36 1902099.

[17] P. Maksymovych, J. Seidel, Y. H. Chu, P. Wu, A. P. Baddorf, L.-Q. Chen, S. V. Kalinin, R. Ramesh, Dynamic conductivity of ferroelectric domain walls in $BiFeO_3$, *Nano Letters* **2011**, *11*, 5 1906.

[18] L. Liu, K. Xu, Q. Li, J. Daniels, H. Zhou, J. Li, J. Zhu, J. Seidel, J.-F. Li, Giant domain wall conductivity in self-assembled $BiFeO_3$ nanocrystals, *Advanced Functional Materials* **2021**, *31*, 1 2005876.

[19] T. Sluka, A. K. Tagantsev, P. Bednyakov, N. Setter, Free-electron gas at charged domain walls in insulating $BaTiO_3$, *Nature Communications* **2013**, *4*, 1 1808.

[20] C. S. Werner, S. J. Herr, K. Buse, B. Sturman, E. Soergel, C. Razzaghi, I. Breunig, Large and accessible conductivity of charged domain walls in lithium niobate, *Scientific Reports* **2017**, *7*, 1 9862.

[21] A. Q. Jiang, W. P. Geng, P. Lv, J.-w. Hong, J. Jiang, C. Wang, X. J. Chai, J. W. Lian, Y. Zhang, R. Huang, D. W. Zhang, J. F. Scott, C. S. Hwang, Ferroelectric domain wall memory with embedded selector realized in $LiNbO_3$ single crystals integrated on Si wafers, *Nature Materials* **2020**, *19*, 11 1188.

[22] H. Lu, Y. Tan, J. P. V. McConville, Z. Ahmadi, B. Wang, M. Conroy, K. Moore, U. Bangert, J. E. Shield, L.-Q. Chen, J. M. Gregg, A. Gruverman, Electrical tunability of domain wall conductivity in $LiNbO_3$ thin films, *Advanced Materials* **2019**, *31*, 48 1902890.

[23] J. A. Mundy, J. Schaab, Y. Kumagai, A. Cano, M. Stengel, I. P. Krug, D. M. Gottlob, H. Doğanay, M. E. Holtz, R. Held, Z. Yan, E. Bourret, C. M. Schneider, D. G. Schlom, D. A. Muller, R. Ramesh, N. A. Spaldin, D. Meier, Functional electronic inversion layers at ferroelectric domain walls, *Nature Materials* **2017**, *16*, 6 622.

[24] D. Meier, J. Seidel, A. Cano, K. Delaney, Y. Kumagai, M. Mostovoy, N. A. Spaldin, R. Ramesh, M. Fiebig, Anisotropic conductance at improper ferroelectric domain walls, *Nature Materials* **2012**, *11*, 4 284.

[25] W. Wu, Y. Horibe, N. Lee, S.-W. Cheong, J. R. Guest, Conduction of topologically protected charged ferroelectric domain walls, *Physical Review Letters* **2012**, *108* 077203.

[26] T. Choi, Y. Horibe, H. T. Yi, Y. J. Choi, W. Wu, S.-W. Cheong, Insulating interlocked ferroelectric and structural antiphase domain walls in multiferroic $YMnO_3$, *Nature Materials* **2010**, *9*, 3 253.

[27] R. G. McQuaid, M. P. Campbell, R. W. Whatmore, A. Kumar, J. M. Gregg, Injection and controlled motion of conducting domain walls in improper ferroelectric Cu-Cl boracite, *Nature Communications* **2017**, *8*, 1 15105.

[28] Y. S. Oh, X. Luo, F.-T. Huang, Y. Wang, S.-W. Cheong, Experimental demonstration of hybrid improper ferroelectricity and the presence of abundant charged walls in $(Ca,Sr)_3Ti_2O_7$ crystals, *Nature Materials* **2015**, *14*, 4 407.

[29] I. Stolichnov, L. Feigl, L. J. McGilly, T. Sluka, X.-K. Wei, E. Colla, A. Crassous, K. Shapovalov, P. Yudin, A. K. Tagantsev, N. Setter, Bent ferroelectric domain walls as reconfigurable metallic-like channels, *Nano Letters* **2015**, *15*, 12 8049.

[30] F. Risch, Y. Tikhonov, I. Lukyanchuk, A. M. Ionescu, I. Stolichnov, Giant switchable non thermally-activated conduction in 180° domain walls in tetragonal $Pb(Zr,Ti)O_3$, *Nature Communications* **2022**, *13*, 1 7239.

[31] X.-K. Wei, T. Sluka, B. Fraygola, L. Feigl, H. Du, L. Jin, C.-L. Jia, N. Setter, Controlled charging of ferroelastic domain walls in oxide ferroelectrics, *ACS Applied Materials & Interfaces* **2017**, *9*, 7 6539, pMID: 28141926.

[32] G. Lindgren, C. Canalias, Domain wall conductivity in $KTiOPO_4$ crystals, *APL Materials* **2017**, *5*, 7 076108.
